# In Silico Structural Modeling and Analysis of Interactions of *Tremellomycetes* Cytochrome P450 Monooxygenases CYP51s with Substrates and Azoles

**DOI:** 10.3390/ijms22157811

**Published:** 2021-07-22

**Authors:** Olufunmilayo Olukemi Akapo, Joanna M. Macnar, Justyna D. Kryś, Puleng Rosinah Syed, Khajamohiddin Syed, Dominik Gront

**Affiliations:** 1 Department of Biochemistry and Microbiology, Faculty of Science and Agriculture, University of Zululand, KwaDlangezwa 3886, South Africa; akapoolufunmilayo@gmail.com; 2 College of Inter-Faculty Individual Studies in Mathematics and Natural Sciences, University of Warsaw, Stefana Banacha 2C, 02-097 Warsaw, Poland; joanna.macnar@student.uw.edu.pl; 3 Biological and Chemical Research Center, Faculty of Chemistry, University of Warsaw, Pasteura 1, 02-093 Warsaw, Poland; juchxd@gmail.com; 4 Department of Pharmaceutical Chemistry, College of Health Sciences, University of KwaZulu-Natal, Durban 4000, South Africa; prosinah@gmail.com

**Keywords:** cytochrome P450 monooxygenases, CYP51, *Tremellomycetes*, sterol 14α-demethylase, *Cryptococcus neoformans*, drug-resistance, web-application, docking

## Abstract

Cytochrome P450 monooxygenase CYP51 (sterol 14α-demethylase) is a well-known target of the azole drug fluconazole for treating cryptococcosis, a life-threatening fungal infection in immune-compromised patients in poor countries. Studies indicate that mutations in CYP51 confer fluconazole resistance on cryptococcal species. Despite the importance of CYP51 in these species, few studies on the structural analysis of CYP51 and its interactions with different azole drugs have been reported. We therefore performed in silico structural analysis of 11 CYP51s from cryptococcal species and other *Tremellomycetes*. Interactions of 11 CYP51s with nine ligands (three substrates and six azoles) performed by Rosetta docking using 10,000 combinations for each of the CYP51-ligand complex (11 CYP51s × 9 ligands = 99 complexes) and hierarchical agglomerative clustering were used for selecting the complexes. A web application for visualization of CYP51s’ interactions with ligands was developed (http://bioshell.pl/azoledocking/). The study results indicated that *Tremellomycetes* CYP51s have a high preference for itraconazole, corroborating the in vitro effectiveness of itraconazole compared to fluconazole. Amino acids interacting with different ligands were found to be conserved across CYP51s, indicating that the procedure employed in this study is accurate and can be automated for studying P450-ligand interactions to cater for the growing number of P450s.

## 1. Introduction

Among fungal diseases, cryptococcosis remains a significant cause of morbidity and mortality in immunocompromised people especially in sub-Saharan Africa [1]. Cryptococcal species such as *Cryptococcus neoformans* and *C. gatti* are primarily responsible for this disease [2]. Across the world, an average of 6% of HIV-infected people have contract this disease, of which 73% of cases were reported in sub-Saharan Africa, with a death rate of 15% [1]. Treatment options for cryptococcosis are limited to only three drugs (amphotericin B, flucytosine, and fluconazole), with few compounds reaching clinical trials [3]. The treatment involves use of an azole drug, fluconazole, as primary or consolidation and maintenance therapy, followed by induction therapy with intravenous amphotericin B with or without flucytosine [4]. In developed countries, patients are initially subjected to induction therapy and then treatment with fluconazole. However, in resource-limited countries such as low- and middle-income countries, treatment is solely based on fluconazole [4,5].

Non-susceptibility of cryptococcal species to fluconazole has been observed around the world and this has become a growing problem [6,7,8,9,10,11]. Interestingly, quite a number of laboratory-based studies reported better performance of other azoles (itraconazole, voriconazole, posaconazole, and isavuconazole) including the new class of tetrazole compound, VT-1129, against cryptococcal species [10,12,13,14,15,16,17,18,19,20,21]. Azole compounds exert their anti-fungal activity by inhibiting the enzyme cytochrome P450 monooxygenase (CYP/P450) CYP51 involved in the synthesis of fungal membrane ergosterol [22,23]. CYP51, also known as sterol 14α-demethylase, is highly conserved across the phyla and stimulates a key enzymatic reaction that involves stereoselective three-step oxidative removal of the 14α-methyl group from the sterol [24]. Because of this important enzymatic reaction, this enzyme has become a drug target against fungal pathogens and protozoan parasites where azole compounds are in use as drugs in clinical practice targeting this enzyme [24,25,26]. A search using the word “CYP51” at Research Collaboratory for Structural Bioinformatics Protein Data Bank (RCSB PDB) [27] indicated the presence of 109 crystal structures (as of 22 January 2021), clearly indicating the enormous importance of this enzyme, especially its exploitation as a drug target [24,28].

Some progress has been made with unraveling the molecular mechanisms responsible for fluconazole non-susceptibility based on CYP51 in cryptococcal species [25,29,30,31,32]. Over-expression of this gene either by sterol regulatory element-binding protein or by virtue of the presence of more than one copy of this gene due to genomic aneuploidy or the mutations in this gene has been to be responsible for fluconazole resistance [25,29,30,31,32]. A point mutation in *CYP51* at G484S resulted in conferring resistance on fluconazole [29]. Azole drug resistance patterns were changed by a single mutation Y145F that caused high fluconazole/voriconazole resistance but increased susceptibility to itraconazole and posaconazole [30]. G344S mutation caused multi-drug resistance to fluconazole, itraconazole, and voriconazole [31]. Detailed information on different mechanisms employed to deal with fluconazole resistance of cryptococcal species was recently reviewed [25].

The *CYP51* gene mutational studies mentioned above are based on sequencing the *CYP51* gene from the fluconazole non-susceptible strains and comparing its sequence with fluconazole-susceptible strains. To date, structure–activity relationship studies on CYP51 from cryptococcal species have not been reported, with the exception of one study published in 2009 reporting the first three-dimensional (3D) model of CYP51 from *C. neoformans*. Based on the in silico results, the authors suggested that G484S substitution changed the orientation of the heme-binding domain, leading to decreased catalytic activity and thus, azole binding conferring the drug resistance [33]. Recently, our laboratory performed genome-wide analysis and annotation of P450s in the fungal class *Tremellomycetes*, which included quite a number of cryptococcal species as well [34]. The study revealed the presence of the *CYP51* gene in all species of *Tremellomycetes*. Considering that structure-activity studies are scarcely reported on CYP51 of cryptococcal species and the availability of a large number of CYP51 sequences [34], this study is aimed at addressing this research gap by performing comparative modeling of CYP51s, which includes assessing their binding patterns to substrates and azoles. CYP51s from other *Tremellomycetes* were included for comparative analysis with CYP51s of cryptococcal species.

## 2. Results and Discussion

### 2.1. CYP51s Grouped as per Tremellomycetes Lifestyle

**Figure 1:**
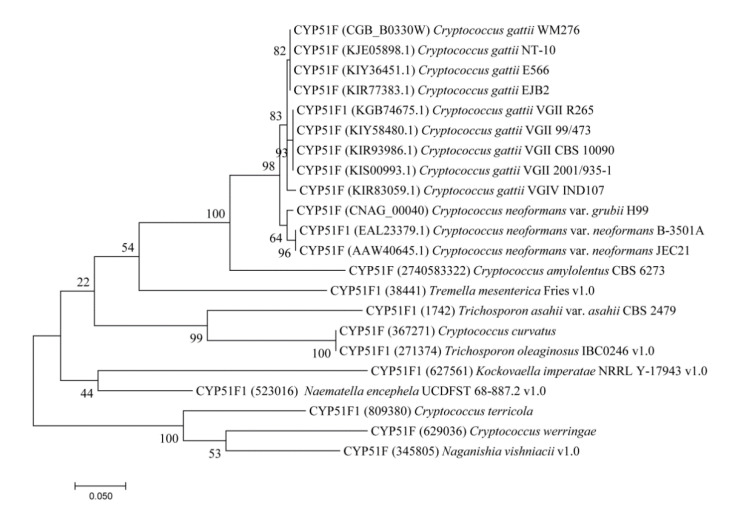
Phylogenetic analysis of CYP51s from Tremellomycetes. The analysis involved 22 CYP51 protein sequences. Phylogenetic analysis was carried out as described in the Methods section and the percentage of trees in which the associated taxa clustered together is shown next to the branches.

Phylogenetic analysis of CYP51s from different *Tremellomycetes* revealed an interesting pattern where CYP51s from species with a similar lifestyle or adaption to similar ecological niches grouped together (Figure 1). CYP51s from human pathogenic species such as *C. neoformans* and *C. gatti* grouped together whereas CYP51s from oleaginous yeasts *C. curvatus* and *Trichosporon oleaginosus* IBC0246 were grouped together with the exception of CYP51 from *C. terricola* (Figure 1). The CYP51s from the *Tremellomycetes* having diverse lifestyles standalone on the tree, indicating considerable changes in CYP51 amino acids after speciation. This phenomenon of grouping P450s from species with similar life style/adaption to similar ecological niches was observed in some bacterial [35,36,37] and fungal species [38,39,40]. This indicates that after speciation, lifestyle/ecological niches played a key role in changing/preserving the amino acid content, as observed for different P450s families in bacteria [35,36,37] and fungi [38,39,40]. 

### 2.2. CYP51s of Tremellomycetes Have All the CYP Characteristic Motifs

Eleven CYP51s from *Tremellomycetes* as representative of diverse lifestyle/ecological niches were selected for structural analysis (Table 1). Three-dimensional structural models were built with template-based modeling using Modeller software version 9.21 [41].

**Figure 2:**
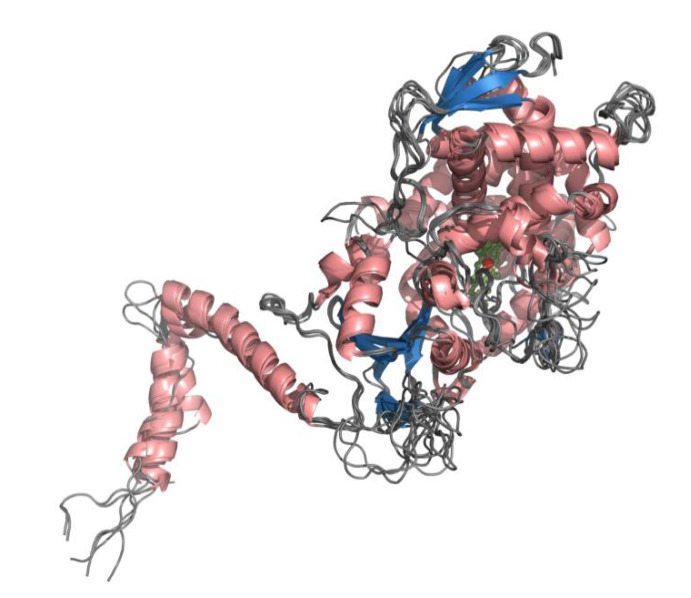
Superimposition of 3D structural models of 11 CYP51s from *Tremellomycetes*. Helices and beta sheets are shown in salmon and sky blue. Heme is shown in green along with the Fe atom in red.

The template 4LXJ (CYP51F from *S. cerevisiae*) [42] was selected as the best template for modeling as the sequence identity with CYP51s from *Tremellomycetes* ranged from 41% to 50% at very high alignment coverage above 90% and the sequence similarity was always well above 70% (Table S1). Multiple sequence alignment of *Tremellomycetes* CYP51s with the template showed very good alignment, with no major deletions when using the template (Supplementary Dataset S1). Three-dimensional modeling of *Tremellomycetes* CYP51s on such good alignment should produce high-quality models as described elsewhere [43]. Furthermore, a point to be noted is that all these CYPs belong to the same subfamily where they share >55% identity criteria to be part of the same subfamily [44]. Considering these facts, the 11 CYP51 models generated in the study, as expected, were found to have highly similar structures with characteristic CYP helices and beta sheets [45,46], with only a few minor differences (Figure 2 and Supplementary Dataset S1). An amphipathic helix and two transmembrane helices are missing from the N-terminal region of CYP51F Cter whereas an amphipathic helix, two transmembrane helices, αA helix and αL helix, and subsequent C-terminal end, are missing from CYP51F1 Nen UCDFST68-887 (protein ID: 523016) (Figure 2 and Supplementary Dataset S1). Lack of these helices may be due to genome editing or gene prediction error where prediction of a complete gene sequence is impossible at present. Compared to the rest of the 10 CYP51s, the αC helix of CYP51F1 TaaCBS2479 is found to be longer with 34 amino acids (Figure 2 and Supplementary Dataset S1). *Tremellomycetes* CYP51s have substrate recognition sites 1–6 (SRS 1-6) with the exception of CYP51F1 Nen UCDFST68-887, where the SRS6 region is missing because the C-terminal region is missing, as described earlier (Figure 2 and Supplementary Dataset 1). Amino acids, part of the SRSs, are highly conserved across the *Tremellomycetes* CYP51s (Supplementary Dataset S1).

### 2.3. Tremellomycetes CYP51s Active Site Cavities Are Highly Hydrophobic

**Figure 3:**
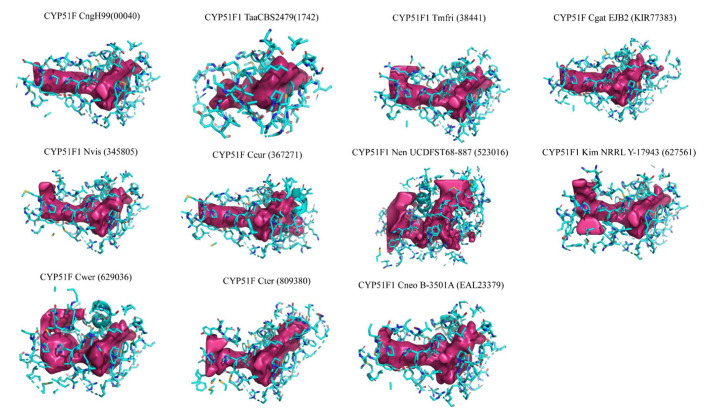
Active site cavities of 11 CYP51s from *Tremellomycetes*. The active site cavity surface is shown in pink and amino acids, part of the active site cavity, are represented using a ball and stick model. A detailed list of amino acids, part of the active site cavity, is presented in Table S2.

Analysis of active site cavities revealed that *Tremellomycetes* CYP51s active site cavities mostly comprise hydrophobic amino acids (Figure 3 and Table S2). The active site cavity volumes range from 685–4173 Å^3^, where CYP51F1 Nen UCDFST68-887 has the largest active site cavity and CYP51F1 TaaCBS2479 has the smallest active site cavity (Figure 3 and Table S2). One of the reasons why CYP51F1 Nen UCDFST68-887 has the largest active site cavity is the opening of a channel right next to heme because of the missing C-terminal region that encompasses SRS6, as described earlier. Thus, the active site cavity volume of this P450 is not an actual volume and a full-length CYP sequence is needed to deduce the correct volume of the active site cavity. CYP51s from prominent human pathogens such as *C. neoformans* var. *grubii* H99, *C. neoformans* var. *neoformans* B-3501A, and *C. gattii* EJB2 have similar sizes to active site cavities (Figure 3 and Table S2). CYP51s from other cryptococcal species have larger active site cavities compared to CYP51s from these three prominent human pathogens (Figure 3 and Table S2). Analysis of the composition of active site cavity amino acids revealed that 50–60% of cavity amino acids are hydrophobic (Table S2), suggesting that *Tremellomycetes* CYP51s’ active site cavities are highly hydrophobic. Among *Tremellomycetes* CYP51s, CYP51F from *C. werringae* has the highest percentage of hydrophobic amino acids (60%). CYP51s active site cavities’ acidic residues range from 1.64–5.73% and the basic residues range from 6.25–12.17%, where CYP51F1 Nen UCDFST68-887 has most acidic amino acids in its cavity and CYP51F from *C. curvatus* has most basic amino acids in its active site cavity (Table S2). The hydrophobic nature of these CYP51s’ active sites is expected considering that they accept highly hydrophobic substrates such as sterols.

### 2.4. A Web Application for Visualization of Tremellomycetes CYP51s Interactions with Ligands

After successful construction of 11 CYP51s 3D models, we then proceeded to understand their interactions with ligands such as substrates and azoles. Among substrates, apart from the fungal CYP51 substrates lanosterol and eburicol [24], we also included the plant CYP51 substrate obtusifoliol [24] as a positive control to assess the accuracy of the study. Among the azoles, five are triazoles (clotrimazole, fluconazole, itraconazole, ketoconazole, and voriconazole) and one is tetrazole (VT_1129). The rationale for including these azoles, especially itraconazole, is to assess if any corroboration occurs between the in silico study and the laboratory studies that showed itraconazole performing better than the fluconazole [10,12,13,14,15,16,17].

**Figure 4:**
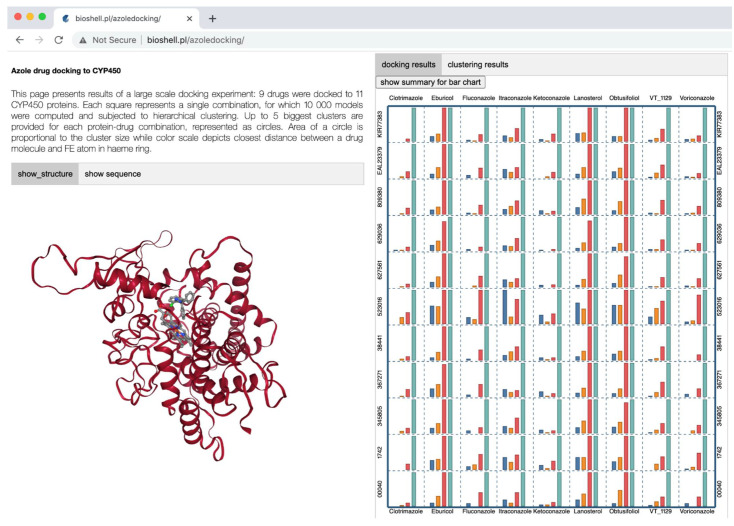
This graphical overview of docking results at online web application. There are 99 squares representing the 11 × 9 = 99 CYP51-ligand combinations considered in this work. CYP51s were indicated with their protein IDs. Each square displays up to four bars, corresponding to models classified as ‘clashes’, ‘correct’, ‘near’, and ‘pocket’ (blue, orange, red, and cyan, respectively). Bars taller than 60 observations were reduced to 60 to fit into a plotting area; actual values are provided in Table 1. Docking results and corresponding information can be accessed at our online web application (Http://Bioshell.Pl/Azoledocking/).

Docking of each CYP51 *vs*. each ligand in 11 × 9 resulted in generating 99 combinations (Figure 4). For each of these combinations, we generated 10,000 models as described in the Methods section. Each of these combinations was analyzed independently. The huge amount of data generated during these docking calculations posed a formidable challenge to the analysis. Therefore, we developed a custom web application for this purpose, and made it publicly available at the address: http://bioshell.pl/azoledocking/. The application can display every model that was generated with its basic numerical parameters, such as Rosetta energy, number of hydrogen bonds, etc. The results can be browsed by cluster, sorted, etc. 

### 2.5. Tremellomycetes CYP51s Have the Highest Preference for Itraconazole

Structures for the complexes (CYP51-ligand) considered in this study have not yet been established experimentally. Therefore, we cannot compare these study results to any reference structure. Nevertheless, the geometry of interaction between a heme moiety of P450s and azole drugs has been studied extensively in P450 enzyme systems where azole drugs are known to coordinate with the ferric core of heme group [47]. A lone pair of aromatic nitrogen of azole rings forms a semi-covalent bond with the Fe atom of length d_NFe_ = 2.1 Å [47]. In our analysis, we report the closest distance between heme’s iron and an aromatic nitrogen atom of an azole drug as a measure of success. For the cases of eburicol, lanosterol, and obtusifoliol, d_NFe_ denotes the closest distance between Fe and any atom of a ligand. Since we allowed Rosetta to sample a wide range of binding spots, we observe ligands located in virtually any region of a receptor. In order to assess the performance of Rosetta *LigandDocking* protocol critically, we included all models in our analysis.

The values of d_NFe_ range from below 2 Å to 80 Å. For the analysis, we defined the following four groups, based on the d_NFe_ distance: Bulleted lists look like this:

- ‘clashes’ where a ligand was closer to the Fe than 1.5 Å;
- ‘correct’ where d_NFe_ was in the range 1.5–2.5 Å;
- ‘near’ with d_NFe_ in the range 2.5–3.5 Å;
- ‘pocket’ with d_NFe_ in the range 3.5–8.0 Å.

The number of models in each of the four categories is provided in Table 1, as well as shown in Figure 4 by blue, orange, red, and cyan bars, respectively. The definition of these categories was based on a visual analysis of the resulting structures using the online application. The distance ranges defining them were manually adjusted to keep the classification meaningful for as many models as possible. Information on each of these four were elaborated in the supplementary information under the title “CYP51-ligand groups”.

Ligand binding affinity under physiological conditions results from free energy gain upon binding. This free energy change includes entropic contributions as well as other effects such as solvent, ions, etc. Although some of these effects (most notably the interactions with solvent) are included in the Rosetta force field as mean field potentials, the energy value reported by Rosetta primarily accounts for conformational energy (i.e., enthalpy). In order to assess relative binding affinity, one has to take into account the number of observations for a particular conformation. The docking procedure that was applied allowed Rosetta to place a ligand virtually anywhere at the protein surface to avoid any conformational bias. The numbers of correctly docked models given in Table 1 therefore provide an estimation of binding affinity for different ligands. Based on these results, it is clear that *Tremellomycetes* CYP51s’ natural substrate, lanosterol, achieved the top score, followed by obtusifoliol and eburicol, indicating that our method of analysis is correct, as one can expect the highest binding affinity to natural substrates. It is also interesting to see the pattern of preference for azoles by *Tremellomycetes* CYP51s where itraconazole is preferred azole followed by VT_1129 and fluconazole, which is the least favored before ketoconazole (Table 1). This preference pattern perfectly matches wet laboratory data where itraconazole and VT_1129 were shown to perform better than fluconazole [10,12,13,14,15,16,17,18,19,20,21]. Overall, based on these results, we can safely say that our method of analysis is correct.

Given the drawbacks of the Rosetta scoring functions as elaborated on the supplementary section “CYP51-ligand”, the chance of selecting a correct complex without prior knowledge of the actual in vivo conformation is low. A commonly used remedy to solve this problem is structural clustering. Thus, in this study, we used this method to select the correct CYP-ligand complex.

**Figure 5:**
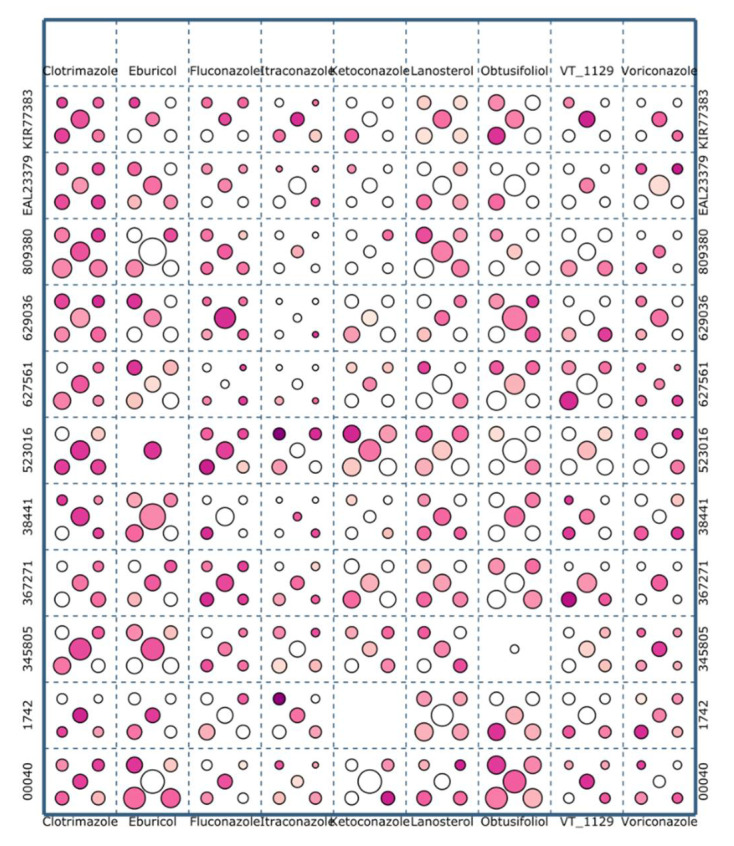
Graphical representation of results of hierarchical clustering. Each of the 99 squares displays up to five clusters, represented by circles. The circular area is proportional to the size of the respective cluster, while the color scale denotes the d_NFe_ distance, with darker colors representing shorter values. All clusters of at least 10 structures found by hierarchical clustering are marked on this figure.

Clustering analysis is a method commonly used in the field of biomacromolecular modeling. It helps to analyze large amounts of data generated by computer simulations and to overcome inaccuracies of molecular force fields. A group of structurally similar conformations corresponds to a single local energy minimum [48]. In general, it is expected that a conformation corresponding to a larger cluster will be more favored entropically. Entropic contributions, necessary to estimate the free energy of a given conformation, are typically not described properly by molecular modeling force fields. Giving preference to larger clusters during the process of selecting the final docking result, implicitly allows inclusion of these entropic contributions. Here, we employed a hierarchical agglomerative clustering algorithm, as described in the Methods section. Following the standard protocols in the field, we report the five largest clusters for every protein–ligand combination. The clusters are indicated graphically in Figure 5 as circles. A circular area is proportional to a cluster size while the color scale represents the d_NFe_ values, in a range from 1 to 80 Å (see Figure 5). In three cases, no suitable clusters were found (Figure 5). All these clustering results can be browsed online at: http://bioshell.pl/azoledocking/.

All the following analyses require selection of a single representative model for each of the 99 modeling cases. The clustering procedure described above is certainly helpful, but on its own, is unable to select the final solution in every case. Therefore, we used a web application (http://bioshell.pl/azoledocking/) to browse the results and manually selected final conformations. In this process, we took into account the classification resulting from the clustering procedure, the chemical correctness of a conformation, and the number of interactions (stacking, H-bonding, and Van der Waals) between a ligand and a protein. The number of such interactions listed in a table of the web application facilitates the selection process.

### 2.6. High Conservation Observed in Tremellomycetes CYP51s Amino Acids Interacting with Ligands

After selection of each of the CYP51-ligand complex as described above, we further analyzed the amino acids interacting with different ligands and their location. This is a very important aspect to check the accuracy of our method employed to dock and select CYP51-ligand complexes. As all of these CYPs belong to the same subfamily and high conservation of amino acids is observed in SRS regions (Supplementary Dataset S1), if our analysis is correct, we should observe high conservation of amino acids interacting with analyzed ligands across the CYPs. As predicted, our analysis revealed conservation of a large number of amino acids interacting with docked ligands across the CYPs (Table 2 and Table S2). Mapping of the amino acids interacting with ≥5 ligands revealed that these amino acids are part of SRSs (Supplementary Dataset S1). We also identified amino acids across the CYPs that are able to form hydrogen bonds with ligands and interact with ligands via stacking and Van der Waals interactions (Table S2). This suggests that our method of docking and selecting the CYP-ligand complexes is correct and in future these aspects can be automated to understand the CYP-ligand interactions to cater for the growing number of CYPs. A point to be noted that CYP51F1 TaaCBS2479 due to its compact active site cavity, has more amino acids interacting with ligands compared CYP51F1 Nen UCDFST68-887 where due to its larger active site cavity ligands are not strongly binding and thus, less number of amino acids found to be interacting with the ligands (Table 2). 

Analysis of amino acid substitutions in CYP51s that are found to be critical in response to different azole drugs [29,30,31] revealed that Y145 and G484 amino acids are absolutely conserved in the *Tremellomycetes* CYP51s (Supplementary Dataset 1 indicated with “X”). However, substitution of glycine at 410 position (formerly reported as G344) [31] with alanine (in three CYP51s), serine (in two CYP51s), lysine (in two CYP51s), and arginine (in a single CYP51) is found (Supplementary Dataset 1 indicated with “X”). This is quite an interesting observation as it has been reported that G344S substitution in six strains of *C. neoformans* var. *grubii* strain, NUBS14020, resulted in the development of multi-drug resistance to azoles such as fluconazole, itraconazole, and voriconazole [31]. Considering these findings, it would be interesting to see the response of the strains containing G344 substitutions in their CYP51s.

## 3. Materials and Methods 

### 3.1. CYP51s Used in the Study

Twenty-one CYP51s from *Tremellomycetes* were used in this study. Among 21 CYP51s, 11 CYP51s from different *Tremellomycetes* representing diverse lifestyles/adaptation to different ecological niches were selected for structural analysis (Table 3). A detailed description of the well-known characteristics of *Tremellomycetes* can be found in a recently published article [34]. All CYP51 sequences from *Tremellomycetes* were retrieved from a recently published article from our laboratory [34] and used in the study. CYP51 sequences used in the study are presented in Supplementary Information. 

### 3.2. Phylogenetic Analysis

Phylogenetic analysis of CYP51s from *Tremellomycetes* was carried out using the maximum likelihood method based on the JTT matrix-based model [49] and 500 bootstrap replications. Analysis and visualization of the phylogenetic tree were carried out using molecular evolutionary genetic analysis [50].

### 3.3. Homology Modeling of CYP51s

A 3D structural model for each CYP51s (Table 3) was constructed by employing a template-based modeling procedure. The template was selected from a non-redundant subset of the Protein Data Bank [51] by searching the database with the HHpred algorithm [52] available on the website. CYP51 from *Saccharomyces cerevisiae* (PDB code: 4lxj) [42] which has the highest E-value, highest percentage identity, and the highest alignment coverage with all 11 CYP51s of *Tremellomycetes* was selected as the best template for modeling of CYP51s. Although some CYP51s share more than 50% identity with other templates, we did not see much added value from using multiple templates. Unfortunately, they will not help in bridging gaps introduced by an alignment. On the other hand, we decided to include a ligand from the template structure in the homology modeling protocol to render the pocket in the active state. The use of multiple templates would considerably have complicated the modeling process. Thus, in this study, we used CYP51 from *S. cerevisiae* as a template. Query to template alignments were also obtained from the HHPred results. Manual editing was introduced when necessary. CYP51s structural models were built based on these alignments with Modeller software (version 9.21) [41]. Three-dimensional modeling of CYP51s was performed based on structural information from the template 4LXJ where heme cofactor and ligand molecules (lanosterol and heme molecule) were explicitly included during the modeling to ensure correct geometry of an active site. The models were checked with Molprobity [NN] which reported 7 to 21 residues (for CYP51F1 Kim NRRL Y-17943 and TaaCBS2479, respectively) outside the allowed Ramachandran region. Detailed statistics are provided in Table S3.

### 3.4. Preparation of CYP51s Models for Docking

Most calculations related to docking were performed with the Rosetta [53] software package, dedicated to biomolecular structure prediction, design, and docking. The software was collaboratively developed by over 60 laboratories from all over the world [54]. The clean-pdb.py script (distributed with the software package) [55] was used to remove all non-protein molecules from the model. Optimization of bonds and dihedral angles was also performed [55]. Rosetta’s molfile_to_param.py script was used to prepare input files: PDB and params (internal Rosetta format) for each ligand, based on the particular SDF files, downloaded from PubChem following the protocol described elsewhere [56], obtained from Rosetta Ligand Docking Tutorial online [55]. Ligand conformers generated with Openbabel [57], and BCL [58] were used, following the protocol used in the Rosetta Community developed at the Meiler’s laboratory. A Rosetta program named ligand_rpkmin was used to minimize protein side chains present near the heme molecule following the protocol used in the Rosetta Community developed by our laboratory. The protein conformation with the lowest energy was chosen for docking procedures.

### 3.5. Ligands and Their Docking Procedure

CYP51 substrates (lanosterol, obtusifoliol, and eburicol), azole drugs such as itraconazole, clotrimazole, voriconazole, fluconazole, and ketoconazole including the novel fungal CYP51 inhibitor VT-1129, were used in the study for assessing their binding pattern with 11 CYP51s of *Tremellomycetes* (Table 3). Chemical structures of the ligands used in the study are presented in Figure S1. In silico docking of these nine ligands to each of the CYP51 3D models was performed. Docking simulations were done with a PyRosetta script [59]. For this purpose, we adapted a standard script distributed with Rosetta Ligand Docking Tutorial [55] and PyRosetta notebook 08.01-Ligand-Docking-XMLObjects [60].

The ligand docking procedure was divided into two stages: low and high resolution. During the first, semi-global stage, a ligand was free to sample the protein surface within a sphere of 20 Å, which covers nearly the whole protein. A soft variant of the Rosetta score function called “ligand_soft_rep” was used at this stage to ease energy barrier crossing. Repulsive LJ term and Coulomb interactions were ramped down while hydrogen bonds were promoted. Any side chain within 6 Å from a ligand was also allowed to change its conformation. During the second, high-resolution stage, HighResDocker mover was used for local refinement of a conformation obtained from the previous stage. A final model was subjected to all-atom energy minimization and scoring. The procedure was repeated independently 10,000 for each ortholog-ligand combination and 10,000 resulting poses were subjected to further analysis.

### 3.6. Hierarchical Agglomerative Clustering

All clustering analyses in this work were performed with the ap_ligand_clustering program of the BioShell 3.0 package [61]. Here, we briefly summarize the procedure as the details of this approach have been reviewed and the software has already been published [62]. The procedure begins with placing every structure in a separate cluster of size 1. Then, at every step, the two closest clusters are found and merged into one. The newly merged cluster contains all structures from its ancestors and the total number of clusters decreases by one. The procedure is repeated until a convergence condition is met. Here, we stop clustering when the distance between the two closest clusters exceeds a certain distance cutoff, e.g., 5.0 Å. There are a few widely used approaches to compute a distance between two clusters. In this work, we assumed the complete link strategy: the distance between two clusters i.e., groups of structures, is defined as the smallest distance possible between any structure from the first group and a structure from the second group. Clustering with complete link strategy results in relatively many small but tight clusters. A single clustering calculation of 10,000 models takes around 5 min on a single CPU core.

It is also very convenient to be able to select a “representative” element of a cluster. Many methods have been devised for this purpose in the literature, e.g., taking an average of all the cluster elements. Averaged conformation, however, may not represent a chemically correct structure. Therefore, we defined a “middle” structure (often named ‘medoid’ in the literature) as the one for which the sum of distances to all other members of the cluster is the smallest.

### 3.7. Protocols

The detailed description of our modeling protocols, comparative modeling, docking, and analysis are given on the laboratory ‘Protocols’ website: https://labnotes.readthedocs.io. The website provides all the necessary files, scripts, and commands that would allow other researchers access to our calculations.

### 3.8. Data Visualization

In order to analyze the results of the calculations extensively and thoroughly, we built a custom visualization tool (http://bioshell.pl/azoledocking/). The application displays plots, statistics, and 3D structural models in a web browser. The server side is run by a Flask application server, also implemented in Python. The client side (i.e., the web browser part) utilizes VisuaLife Python library (visualife.readthedocs.io/) to render plots, charts, and tables. NGL biomolecular viewer [63] is employed to display structural models.

### 3.9. Active Site Cavity and Ligand Interacting Amino Acids Detection

Active site cavities in CYP51 models were detected using CavityPlus [64]. Interactions between a ligand and the protein were detected automatically with a BioShell package [61]. Van der Waals interactions were assigned when any atoms of two residues (including hydrogens) were closer to each other than the sum of their VdW radii + 0.5 Å. Hydrogen bonds were assigned when the distance between the donated hydrogen atom and its acceptor was shorter than 3 Å and the planar angle formed by the three atoms, pre-acceptor, acceptor, and the hydrogen was wider than 90 deg. Stacking interactions were recorded when the distance between the two aromatic ring centers was shorter than 7 Å.

## 4. Conclusions

Cytochrome P450 monooxygenases (CYPs/P450s) are some of the well-studied enzymes in the field of biology. Among CYPs, CYP51 serves as drug target against fungal pathogens. In the case of cryptococcosis, a life-threatening fungal disease in immunocompromised patients, studies indicated that cryptococcal species are developing resistance to the currently used azole drug fluconazole. Despite knowing that CYP51 is the prime target of this drug, mutations in this gene conferring resistance on fluconazole and a large number of in vitro studies indicating that other azoles perform better than fluconazole, to date very few studies on CYP51s structure from cryptococcal or other *Tremellomycetes* and their interactions with azoles have been reported. To address this research gap, in this study, we performed comparative modeling of CYP51s and their interactions with different ligands from *Tremellomycetes*. Phylogenetic analysis of CYP51s indicated some kind of amino acid conservation after speciation, as CYP51s grouped as per their species lifestyle/ecological niches. Structural models built for 11 CYP51s revealed the presence of P450 characteristic motifs and high conservation of amino acids in substrate recognition sites (SRSs) 1–6. The active site cavities of CYP51s were found to be highly hydrophobic to accommodation of hydrophobic sterol substrates. Since the structures for the complexes (CYP51-ligand) we considered have not yet been established experimentally, we performed global docking of ligands (three substrates and six azoles) with 10,000 combinations for each of the complex and the correct complexes were selected by hierarchical agglomerative clustering using the cluster program of the BioShell package. To analyze the huge data generated in this study, we developed an online visualization tool (http://bioshell.pl/azoledocking/). These study results indicated that *Tremellomycetes* CYP51s have the highest preference for its natural substrate lanosterol, followed by obtusifoliol and eburicol. Among azoles, *Tremellomycetes* CYP51s have the highest preference for itraconazole, followed by VT_1129, clotrimazole, voriconazole, fluconazole, and ketoconazole. *Tremellomycetes* CYP51s’ high preference for itraconazole is possibly the reason why this azole perform better than fluconazole, as observed in a large number of in vitro studies. The methodology followed in this study is correct, as the amino acids we identified as interacting with different ligands are highly conserved across the CYP51s, indicating no errors in the procedure. Furthermore, most of these amino acids were found to be part of SRSs. In this study, we focused on a single subfamily of CYPs simply to assess the methods employed in the study, but this method needs to be validated for the analysis of different CYP families and their interactions with different ligands. Studies are in progress to develop a website on which this program will be automated to study CYPs’ interactions with different compounds. This type of dedicated website for this family of enzymes is desperately needed, as the number of CYPs is growing exponentially every day. The results of this study further strengthen the previous laboratory results that itraconazole and VT_1129 will be potential compounds in the fight against cryptococcosis and their efficacy against cryptococcal species needs to be determined. One of the interesting results from this study is the identification of non-conservation of G344 amino acid in *Tremellomycetes* CYP51s, including in some cryptococcal species. It would be interesting to see if these *Tremellomycetes* are resistant to fluconazole, itraconazole, and voriconazole, as was reported for G344S substituted species.

## Figures and Tables

**Table 1:** Analysis of *Tremellomycetes* CYP51s and ligand complexes. Number of models falling into ‘clashes’, ‘correct’, ‘near’, and ‘pocket’ group summarized by ligands. These numbers are summed up over all 11 proteins, so the total number of models for each inhibitor is 99,000.

Ligand	No. Clashes	No. Correct	No. Near	No. Pocket
Lanosterol	131	212	809	12,032
Obtusifoliol	116	209	720	11,670
Eburicol	116	202	828	12,511
Itraconazole	185	108	256	9041
average	76	98	387	7555
VT_1129	24	76	250	4552
Clotrimazole	1	25	98	2825
Voriconazole	21	22	195	5020
Fluconazole	42	19	240	5040
Ketoconazole	55	14	87	5307

**Table 2:** Analysis of *Tremellomycetes* CYP51s amino acids interacting with different ligands. Amino acids interacting with ≥5 ligands are presented in the table. Amino acids are colored based on their interactions with nine ligands (red), eight ligands (blue), seven ligands (brown), six ligands (green), and five ligands (pink). A complete list of amino acids interacting with different ligands is listed in Table S2.

CYP51F	Amino Acids Interacting with Ligands (≥5 Ligands)
CYP51F CngH99	F240, Y145, A317, F139, I386, M528, T321, V144, Y131
CYP51F1 TaaCBS2479	S159, A158, A334, F127, F257, H337, I403, M333, M545, P402, S157, T123, T338, T544
CYP51F1 Tmfri	A307, F126, F228, I376, M521, T311, V131, Y118, Y132
CYP51F Ccur	A307, F128, F230, I376, M518, T124, T311, Y120, Y134
CYP51F1 Nvis	F228, Y120, A305, F128, I374, T309, Y134
CYP51F1 Nen UCDFST68-887	A210, A214, F137, F35, H217, I283, T218, T31, V40, Y27, Y41
CYP51F1 Kim NRRL Y-17943	A313, F235, H316, I382, L129, M526, T130, T317, T525, Y126, Y140
CYP51F Cwer	A304, F128, F227, I373, S375, T124, T308, Y120, Y134
CYP51F Cter	A237, F160, F60, H240, L306, L55, M236, M451, T241, T56, Y52, Y66
CYP51F1 Cneo B-3501A	F234, I380, A311, F133, H314, I523, M310, T129, T315, V524, Y125, Y139
CYP51F Cgat EJB2	F240, A317, F139, I386, M316, M528, T135, T321, Y131, Y145

**Table 3:** Information on CYP51s from *Tremellomycetes* used for structural analysis in this study. Amino acid sequences of CYP51s have been retrieved from the published data [34] and used in the study. The databases used for collecting CYP51 sequences were presented in parenthesis under the P450 ID column.

Species Name	Species Abbreviation	CYP ID	CYP Abbreviation Used in the Study
*Cryptococcus neoformans* var. *grubii* H99	CngH99	00040 (NCBI)	CYP51F CngH99
*Trichosporon asahii* var. *asahii* CBS 2479	TaaCBS2479	1742 (JGI)	CYP51F1 TaaCBS2479
*Tremella mesenterica* Fries v1.0	Tmfri	38441 (JGI)	CYP51F1 Tmfri
*Cryptococcus curvatus*	Ccur	367271 (JGI)	CYP51F Ccur
*Naganishia vishniacii* v1.0	Nvis	345805 (JGI)	CYP51F1 Nvis
*Naematella encephela* UCDFST 68-887	Nen UCDFST68-887	523016 (JGI)	CYP51F1 Nen UCDFST68-887
*Kockovaella imperatae* NRRL Y-17943	Kim NRRL Y-17943	627561 (JGI)	CYP51F1 Kim NRRL Y-17943
*Cryptococcus werringae*	Cwer	629036 (JGI)	CYP51F Cwer
*Cryptococcus terricola*	Cter	809380 (JGI)	CYP51F Cter
*Cryptococcus neoformans* var. *neoformans* B-3501A	Cneo B-3501A	EAL23379 (NCBI)	CYP51F1 Cneo B-3501A
*Cryptococcus gattii* EJB2	Cgat EJB2	KIR77383 (NCBI)	CYP51F Cgat EJB2

Abbreviations: NCBI, National Center for Biotechnology Information; JGI, Joint-Genome Institute, USA.

## Data Availability

Data is contained within the article or supplementary material.

## Supplementary Materials

The following are available online at https://www.mdpi.com/article/10.3390/ijms22157811/s1, Reference [42] is cited in the Supplementary Materials.
