# Supplementary material for: In Silico Structural Modeling and Analysis of Interactions of Tremellomycetes Cytochrome P450 Monooxygenases CYP51s with Substrates and Azoles
Source: Int J Mol Sci. 2021 Jul 22;22(15):7811. doi: 10.3390/ijms22157811 (PMC8346148; doi:10.3390/ijms22157811)
Supplement: Supplementary file 1 [file ijms-22-07811-s001.zip › Supplementary Information/Supplementary Dataset 1.pdf]

Article

In silico Structural Modeling and Analysis of Interactions of *Tremellomycetes* Cytochrome P450 Monooxygenases CYP51s with Substrates and Azoles

Olufunmilayo Olukemi Akapo [1,†], Joanna M. Macnar [2,3,†], Justyna D. Kryś [3], Puleng Rosinah Syed [4], Khajamohiddin Syed [1,†], Dominik Gront [3,*]

1 Department of Biochemistry and Microbiology, Faculty of Science and Agriculture, University of Zululand, KwaDlangezwa 3886, South Africa; akapoolufunmilayo@gmail.com (O.O.A); khajamohiddinsyed@gmail.com (K.S.)

2 College of Inter-Faculty Individual Studies in Mathematics and Natural Sciences, University of Warsaw, Stefana Banacha 2C, 02-097 Warsaw, Poland; joanna.macnar@student.uw.edu.pl (J.M.M.)

3 Faculty of Chemistry, Biological and Chemical Research Center, University of Warsaw, Pasteura 1, 02-093 Warsaw, Poland; juchxd@gmail.com (J.D.K); dgront@gmail.com (D.G);

4 Department of Pharmaceutical Chemistry, College of Health Sciences, University of KwaZulu-Natal, Durban 4000, South Africa; prosinah@gmail.com (P.R.S.)

† Authors contributed equally to the work

* Correspondence: khajamohiddinsyed@gmail.com (K.S.) and dgront@gmail.com (D.G.)

Supplementary Dataset 1: Structural alignment of *Tremellomycetes* CYP51s with template 4LXJ. P450 characteristic notation for α-helices and β strands and substrate recognition sequences (SRS) were mapped based on the template. Residues interacting with ≥5 ligands highlighted in yellow. Residues playing a role in azole drug resistance are indicated with "X".

```
                amphipathic helix                    transmembrane helix (TM)
4lxj
              1         10                    20           30                40
4lxj      ..SIVGEALEYV......NIGLSHFLALPLAQRISLII....IIPFIYNIVWQLLYSLRK
629036    .............QSILLGFLPTLFSSFGTIGSVLLLLLSLPVLLVAGNVFRQKVLPRDP
345805    ...........MSSSRILSLLPLEQLSWASIA..AIILVGLPALLVVGNIIRQKVLPKDP
809380    ............................................................
627561    .....TEAQREFARNFREGKIQPIDPT..IPLYILSSIAILLFTVVGSNLMQQFLAPRDP
38441     .............AS.VP.WAPQWLLSLPSPLLVLLGIIGFPTLIIFLNVFRQLVLPRDK
1742      ............MSI.VQ.GAIEAALALPLAQKLAAVFIGLPVLAIVINVLSQLLLPKDP
KIR77383  MSAIIPQVQQLLGQL.AQ.YIPPWFTALPTSLKIVIAVIGIPAFIIGLNVFQQLCLPRTK
00040     MSAIIPQVQQLLGQV.AQ.FIPPWFAALPTSVKVVIAVIGIPALVICLNVFQQLCLPRRK
EAL23379  ......QVQQLLGQV.AQ.FFPPWFAALPTSLKVAIAVVGIPALIIGLNVFQQLCLPRRK
271374    ...........MAMD.PS.AVLDWAHALPLPVKIVGAVIGVPLLVIVLNVLRQLIVPTDP
367271    ...........MAMD.PS.AVLDWAHALPLPVKIVGAVIGVPLLVIVLNVLRQLIVPTDP
523016    ............................................................

                                  TM        αA          β1_1    β1_2     αB
4lxj                                                        TT
          50            60          70          80          90         100
4lxj      DRPPLVFYWIPWVGSAVVYGMKPYEFFEECQKKYGDIFSFVLLGRVMTVYLGPKGHEFVF
629036    TLPPLVFHYIPWFGSAATYGMDPYKFLFDCREQYGDLFTFILLGRKMTVALGPKGNNLIL
345805    SLPPVVFHLIPWFGSAATYGMDPYKFLFDCREQYGDLFTFVLLGRKMTVALGPKGNNLIL
809380    .....................DPYKFMFGCRAKYGDLFTFILMGREMTVALGPKGNNLIL
627561    SHPPVVLHYFPWFGSAYSYGTDPYKFLFKCRAKYGDVFTFIMFGRKMTVALGPKGNNLSL
38441     NLPPVVFHYIPWFGSAAYYGEDPYKFYFECREKYGDVFTFVLLGRNITVALGPKGNNLSL
1742      TLPPVVFHVIPWFGSAAAYGMDPYKFLFDCREKYGDVFTFILLGRRMTVALGPKGNNLSL
KIR77383  DLPPVVFHYIPWFGSAAYYGEDPYKFLFECRDKYGDLFTFILMGRRITVALGPKGNNLSL
00040     DLPPVVFHYIPWFGSAAYYGEDPYKFLFECRDKYGDLFTFILMGRRVTVALGPKGNNLSL
EAL23379  DLPPVVFHYIPWFGSAAYYGEDPYKFLFECRDKYGDLFTFILMGRRITVALGPKGNNLSL
271374    TAPPVVFHYIPWFGSAAYYGMDPYKFMFECRDKYGDVFTFILLGRRMTVALGPKGNNLSL
367271    TAPPVVFHYIPWFGSAAYYGMDPYKFMFECRDKYGDVFTFILLGRRMTVALGPKGNNLSL
523016    ..............................................MTVALGPKGNNLSL

                 β1_5     αB_1                                 αC
4lxj                                   TT            ....................
         110        120         130          140
4lxj      NAKLADVSAEAAYAHLTTPVFGKGVIYDCPNSRLMEQKKF....................
629036    GGKLSQVSAEDAYTHLTTPVFGKGVVYDCPNEMLMQQKRM....................
345805    GGRLNQVSAEEAYTHLTTPVFGEGVVYDCSNDLLMQQKRM....................
809380    GGKLSQVSAEEAYTHLTTPVFGKGVVYDCPNDMLMQQKRM....................
627561    GGKVSHVSAEDAYTHLTTPVFGKGVVYDCPNEMLMQQKKF....................
38441     GGKTTQVSAEEAYKHVTTPVFGKGVVYDCPNEMLMQQKKF....................
1742      GGKITHVSAEEAYTHLTTPVFGKGVVYDCPNDMLMQQKKFSRSRVCCFCRSASGSPMPRS
KIR77383  GGKISQVSAEEAYTHLTTPVFGKGVVYDCPNEMLMQQKKF....................
00040     GGKISQVSAEEAYTHLTTPVFGKGVVYDCPNEMLMQQKKF....................
EAL23379  GGKISQVSAEEAYTHLTTPVFGKGVVYDCPNEMLMQQKKF....................
271374    GGKVSHVSAEDAYTHLTTPVFGKGVVYDCPNDMLMQQKKF....................
367271    GGKVSHVSAEDAYTHLTTPVFGKGVVYDCPNDMLMQQKKF....................
523016    GGKVSQVSAEDAYTHLTTPVFGKGVVYDCPNDLLMQQKKF....................
                       SRS1          X

                                          αD                      β3_1
4lxj      ..............
                        150         160         170         180         190
4lxj      ..............VKGALTKEAFKSYVPLIAEEVYKYFRDSKNFRLNERTTGTIDVMVT
629036    ..............VKFGLSTENFRVYVPLIKQETLDFLGKDLKPS.NE..WKGFNALHA
345805    ..............VKFGLSTENLRAYVGMITEETLGFLTNELKASPTA..WQGFDALNA
809380    ..............VKFGLSTENLKSYVPKITRETLDFLSHGLHTSATK..WQSFNALDK
627561    ..............IKHGLTPQAFQTYAPLIWQETHQFFEQEKGFN.VYPGPKSYEAIKF
38441     ..............IKNGLSTEALASYPPLINAECEHYFEKELGFSPTSPGPKTIEVFKI
1742      TPARTFRRNPTNAQIKHGLTTEALSSYAELMRGETRQYFRDHVVT......GKPFEVLEM
KIR77383  ..............IKSGLTTESLQSYPPMITSECEDFFTKEVGISSQKPS.ATLDLLKS
00040     ..............IKSGLTTESLQSYPPMITSECEDFFTKEVGISPQKPS.ATLDLLKS
EAL23379  ..............IKSGLTTESLQSYPPMITSECEDFFTKEVGISPQKPS.ATLDLLKA
271374    ..............IKHGLTTEALQSYASLMPAECHGYFNNELKITKANPGPKTVDVLHV
367271    ..............IKHGLTTEALQSYASLMPAECHGYFNNELKITKANPGPKTVDVLHV
523016    ..............IKHGLTTEALSSYAPLMYKEATDFFEHELGLTPATPGPKKLEALKI

              αE          αF'         αF        α310F''
4lxj
             200         210         220         230         240         250
4lxj      QPEMTIFTASRSLLGKEMRAKLDTDFAYLYSDLDKGFTPINFVFPNLPLEHYRKRDHAQK
629036    MAELTILTASATLQGKEVRGKLDKTFAKRYEALDGGFTPINFMFPNLPLPSYKRRDKAQA
345805    MSELTILTASRTLQGKEVRAGLDKTFAERYEHLDGGFTPINFMFPNLPLPSYRRRDKAQK
809380    LAELITLTASDCLQGREVRAGLDKTFAKRIEALDKGFTPMNFVFPNLPLPAYWNRDKAQK
627561    FSELIIILTASRTLQGKEVRAGLTSSAAKHFEHLDKGFTPINFLFPNLPLPSYRKRDKAQK
38441     MSQLIIILSASRTLQGKEVRESLDSKFAEYFHDLDGGFTPLNFMFPNLPLPSYRKRDKAQK
1742      MQQLIIILTASRTLQGKEVRENLDIRFAKLLEDLDKGFTPVNFLFPNLPLPSYKRRDKAQK
KIR77383  MSELIIILTASRTLQGKEVRESLNGQFAKYYEDLDGGFTPLNFMFPNLPLPSYRRRDEAQK
00040     MSELIIILTASRTLQGKEVRESLNGQFAKYYEDLDGGFTPLNFMFPNLPLPSYKRRDEAQK
EAL23379  MSELIIILTASRTLQGKEVRESLNGQFAKYYEDLDGGFTPLNFMFPNLPLPSYKRRDEAQK
271374    MAELIVLTASRTLQGKEVRENMSIRFAKLLEDLDKGFTPLNFMFPNLPLPSYRRRDLAQK
367271    MAELIVLTASRTLQGKEVRENMSIRFAKLLEDLDKGFTPLNFMFPNLPLPSYRRRDLAQK
523016    MSELIIILTAGRSLQGKEVRASLNARFASLYEDLDGGFTPLNFMFPNLPLPSYRRRDRAQK
                                          SRS2                     SRS3
```

```
             αG                                     αH                      αI
4lxj                                                       TT
                 260          270           280           290           300           310
4lxj         AISGTYMSLIKERRKNNDIQ..DRDLIDSLMKNSTYKDGVKMTDQEIANLLIGVLMGGQH
629036       EMSEFYQSIIRGRREGASEH..DHDMIAA.LMASTYRDGTPLSDSDIAHMMIALLMAGQH
345805       AMSEFYQGIIRKRREGTHDH..EYDMISA.LQSSKYKDGTPLSDRDIAHMMIALLMAGQH
809380       EMSEFYQEIIRKRREGTHDH..EHDMLEA.LMGSSYKNGTPLTDSDIAHMMIALLMAGQH
627561       AMSDFYLEIMRKRREGESDV.DEHDMIAA.LTGNEYKDGTPLTDRDVAHMMIAILMAGQH
38441        SMSDFYLNIMQKRREGATDVNGSFDMLAA.LQGCVYRNGVPLTDRDIAHMMIAILMAGQH
1742         EMSDFYMSIIEKRRSGEHDH..ENDMIAA.LQGSVYKNGVPLSDRDISHIMIAILMAGSH
KIR77383     AMSDFYLKIMENRRKGESDH..EHDMIEN.LQGCKYRNGVPLSDRDVAHIMIALLMAGQH
00040        AMSDFYLKIMENRRKGESDH..EHDMIEN.LQSCKYRNGVPLSDRDIAHIMIALLMAGQH
EAL23379     AMSDFYLKIMENRRKGESDH..EHDMIEN.LQSCKYRNGVPLSDRDIAHIMIALLMAGQH
271374       EMSDFYMSIMAKRRTGEHDH..EPDMIQA.LQGSVYRNGTPLSDRDIAHMMIALTMAGQH
367271       EMSDFYMSIMAKRRTGEHDH..EPDMIQA.LQGSVYRNGTPLSDRDIAHMMIALTMAGQH
523016       EMSDFYLDIIRKRREGETEP..EHDMISV.LSGCVYRDGTPLTDRDVSHMMIAILMAGQH

                                          αJ              η1      αJ_1        αK
4lxj
                       320        330           340             350         360
4lxj         TSAATSAWILLHLAERPDVQQELYEEQMRVL...DGGKKELTYDLLQEMPLLNQTIKETL
629036       TSSATSSWTLLHLADRTDVYAELYEEQKEKFGNPDGTFRDMTYEDLKDLPKLDSVIRETL
345805       TSSATSSWTLLHLAERTDIWEELYREQKNKFGNPDGTFRDLTYEELKELPVLDHVIRETL
809380       TSSATSSWTLLHLAQRHDIYEALYQEQKEKFGNADGTFRDLTYEDLKDLPLLDSCIRETL
627561       TSSATSSWTLLHLAQKPLIFKALFEEHRDLFQNEDGTWEDVTYESTKEMPLMAAVIRETL
38441        TSSATSSWALLHLAHRPDVADALYEEQKTLFGNPDGSFRPVEYGDEKRMTLMNSIIRETL
1742         TSSATSSWFLLHLAYDQELQQALYDEQVQLFGNADGSFREMTLEDTRELPLMTACIRETL
KIR77383     TSSATSSWTLLHLADRPDIVEALYQEQKEKLGNPDGTFRDYKYEDLKELPIMDSIIRETL
00040        TSSATSSWTLLHLADRPDVVEALYQEQKQKLGNPDGTFRDYKYEDLKELPIMDSIIRETL
EAL23379     TSSATSSWTLLHLADRPDVVEALYQEQKQKLGNPDGTFRDYRYEDLKELPIMDSIIRETL
271374       TSSATSSWFLLHLAHDQDIQKRLYEEQVEYFGNPDGTFRPMTYEDTKKLPLMDSCIRETL
367271       TSSATSSWFLLHLAHDQDIQKRLYEEQVEYFGNPDGTFRPMTYEDTKKLPLMDSCIRETL
523016       TSSATSSWILLHLAHRPDVVEALYEEQKKLFGNQDGTFREVTYESTKQMTLMEAIIRETL

                           β1_4    β2_1                   β2_2      β1_3    αK_1
4lxj                                    TT                     TT
             370           380          390                   400         410
4lxj         RMHHPLHSLFRKVMKDMHVPN..........TSYVIPAGYHVLVSPGYTHLRDEYFPNAH
629036       RMHAPIHSIMRKVMTDMPVPNALAAPTANEKSSYIVPKGHFVLASPGVAQMDPLIWSDAS
345805       RLHAPIHSIMRKVISDIPVPNTLSSPKQHENATYVIPKGHFVLASPGVAQMDPLIWQNAE
809380       RLHAPLHSIMRKVISDIPVPNSLSSPSQTNDASYVIPKGHFLMACPGVSQMDPLIWQDST
627561       RMHAPIHSIYRKVLQDLEVPPALAAGGN.GSRPYVVPKGHFVVAAPGVSAMDPKVWPDAD
38441        RIHAPIHSIYRKVISPMPVPASLSAPS..ESATYIIPKNYYIVAAPGVSAMDPRIWKDAN
1742         RLHAPIHSIYRKVMLDIAVPPSLAAPS..KDGTYVIPKGHFIVAAPGVSQMDPRIWKDAK
KIR77383     RMHAPIHSIYRKVLSDIPVPPSLAAPS..ENGQYIIPKGHYIMAAPGVSQMDPRIWQDAK
00040        RMHAPIHSIYRKVLSDIPVPPSLSAPS..ENGQYIIPKGHYIMAAPGVSQMDPRIWQDAK
EAL23379     RMHAPIHSIYRKVLSDIPVPPSLSAPS..ENGQYIIPKGHYIMAAPGVSQMDPRIWQDAK
271374       RLHAPIHSIYRKVLQPIVVPQSLAAPS..EDKAYVIPKGNFIVAAPGVSQMDPKIWDDAP
367271       RLHAPIHSIYRKVLQPIVVPQSLAAPS..EDKAYVIPKGNFIVAAPGVSQMDPKIWDDAP
523016       RLHAPIHSIYRKVLSDIPVPASLSSPS..ESSAYVIPEGHFIVAAPGVSQMDPLIWPDAK
                                              X

                 meander_loop                                         η3
4lxj                                                               TT
             420           430                 440          450         460         470
4lxj         QFNIHRWNNDSAS......SYS..VGEEVDYGFGAISKGVSSPYLPFGGGRHRCIGEHFA
629036       IWNPHRWTDEKGMASQALEEYT..SGDKVDYGYGSVSKGTESPYQPFGAGRHRCIGETFA
345805       EWNPHRWDDESGVAAMAAEQYT..EGDKVDYGYGSVSKGTESPYQPFGAGRHRCIGESFA
809380       TWNPYRWSDERGVAAQAVEEYQGEKGEKVDYGFGSVSKGTESPYQPFGAGRHRCIGESFA
627561       KWDPFRWLEDKGMAQEALDSYSGATSEQIDYGFGQVSKGTESPYMPFGAGRHRCVGEQFA
38441        VWEPFRWSDEKGTAAMALNEYTT.GGEKMDYGFGQVSKGTESPYQPFGAGRHRCVGEAFA
1742         TWRPRRWLEEGGVANAANEEYTS..GERVDYGFGAVSKGTESPYQPFGAGRHRCVGEQFA
KIR77383     VWNPARWHDEKGFAAAAMAQYTK..AEQVDYGFGSVSKGTESPYQPFGAGRHRCVGEQFA
00040        VWNPARWHDEKGFAAAAMAQYSK..AEQVDYGFGSVSKGTESPYQPFGAGRHRCVGEQFA
EAL23379     VWNPARWHDEKGFAAAAMVQYTK..AEQVDYGFGSVSKGTESPYQPFGAGRHRCVGEQFA
271374       RWNPLRWLVDGGIAKTANEQYQA..GEKVDYGFGAVSKGTESPYQPFGAGRHRCIGEQFA
367271       RWNPLRWLVDGGIAKTANEQYQA..GEKVDYGFGAVSKGTESPYQPFGAGRHRCIGEQFA
523016       TWNPYRWLDEKGVAASAKEQYSGATSEQVDYGFGQVSKGTESPYQPFGAGRHR.......
                                                                X

                 αL        β3_3                          β3_2
4lxj                                 TT                                TT
                         480          490           500          510          520          530
4lxj         YCQLGVLMSIFIRTLKWHYPEG.KTVPPPDFTSMVTLPTGPAKIIWEKRNPEQKIGGRHH
629036       YLQLQTILATFIRNVEMKLD...GPFK.TNYQTMVVLPLPGTKIL..YRNRK........
345805       YVQLQTILATLCRRVVLKLE...GPFPAPNYQTMIVLPLKGQTKI.MYKLRA........
809380       YVQLQTIIATVVRNLTLKFE...GDFPSPNYQTMVVLPKGGEIL...FKSRQ........
627561       YLQLSVIMSYIVRNYDIQLL...GAFPQTNYNTMIVLPLNGYVTF.KKRGQSKWI.....
38441        YVQLSVIIAYIIRNYSLRLETPDGLFPKTNYRTMIVLPLNGLMSL.TKRKQE........
1742         YLQLTMLVSEIIRTFKVEPGAP..QFPETNYQTMIVLPLHGMIKL.EPRK..........
KIR77383     YTQLSTIFTYVVRNFTLKLAVP..KFPETNYRTMIVQPTNPLVTF.TLRNAEVK......
00040        YTQLSTIFTYVVRNFTLKLAVP..KFPETNYRTMIVQPNNPLVTF.TLRNAEVK......
EAL23379     YTQLSTIFTYVVRNFTLKLAVP..KFPETNYRTMIVQPNNPLVTF.TLRNAEVKQEV...
271374       YLQLTLVVGEVVRNYKLTAAQR..EFPKTNYQTMIVLPLDPKITF.EARV..........
367271       YLQLTLVVGEVVRNYKLTAAQR..EFPKTNYQTMIVLPLDPKITF.EARV..........
523016       ............................................................
```
