# Supplementary material for: In Silico Structural Modeling and Analysis of Interactions of Tremellomycetes Cytochrome P450 Monooxygenases CYP51s with Substrates and Azoles
Source: Int J Mol Sci. 2021 Jul 22;22(15):7811. doi: 10.3390/ijms22157811 (PMC8346148; doi:10.3390/ijms22157811)
Supplement: Supplementary file 1 [file ijms-22-07811-s001.zip › Supplementary Information/Supplementary Information.pdf]

Article

In silico Structural Modeling and Analysis of Interactions of *Tremellomycetes* Cytochrome P450 Monooxygenases CYP51s with Substrates and Azoles

Olufunmilayo Olukemi Akapo [1,†], Joanna M. Macnar [2,3,†], Justyna D. Kryś [3], Puleng Rosinah Syed [4], Khajamohiddin Syed [1,*] and Dominik Gront [3,*]

1 Department of Biochemistry and Microbiology, Faculty of Science and Agriculture, University of Zululand, KwaDlangezwa 3886, South Africa; akapoolufunmilayo@gmail.com
2 College of Inter-Faculty Individual Studies in Mathematics and Natural Sciences, University of Warsaw, Stefana Banacha 2C, 02-097 Warsaw, Poland; joanna.macnar@student.uw.edu.pl
3 Biological and Chemical Research Center, Faculty of Chemistry, University of Warsaw, Pasteura 1, 02-093 Warsaw, Poland; juchxd@gmail.com
4 Department of Pharmaceutical Chemistry, College of Health Sciences, University of KwaZulu-Natal, Durban 4000, South Africa; prosinah@gmail.com
* Correspondence: khajamohiddinsyed@gmail.com (K.S.); dgront@gmail.com (D.G.)
† Authors contributed equally to the work.

CYP51 sequences used in this study

>CYP51F(CNAG_00040/Q09GQ2)Cryptococcus neoformans var. grubii H99

MSAIIPQVQQLLGQVAQFIPPWFAALPTSVKVVIAVIGIPALVICLNVFQQLCLPRRKDL

PPVVFHYIPWFGSAAYYGEDPYKFLFECRDKYGDLFTFILMGRRVTVALGPKGNNLSLGG

KISQVSAEEAYTHLTTPVFGKGVVYDCPNEMLMQQKKFIKSGLTTESLQSYPPMITSECE

DFFTKEVGISPQKPSATLDLLKSMSELIILTASRTLQGKEVRESLNGQFAKYYEDLDGGF

TPLNFMFPNLPLPSYKRRDEAQKAMSDFYLKIMENRRKGESDHEHDMIENLQSCKYRNGV

PLSDRDIAHIMIALLMAGQHTSSATSSWTLLHLADRPDVVEALYQEQKQKLGNPDGTFRD

YKYEDLKELPIMDSIIRETLRMHAPIHSIYRKVLSDIPVPPSLSAPSENGQYIIPKGHYI

MAAPGVSQMDPRIWQDAKVWNPARWHDEKGFAAAAMAQYSKAEQVDYGFGSVSKGTESPY

QPFGAGRHRCVGEQFAYTQLSTIFTYVVRNFTLKLAVPKFPETNYRTMIVQPNNPLVTFT

LRNAEVK

>CYP51F1(1742)Trichosporon asahii var. asahii CBS 2479

MSIVQGAIEAALALPLAQKLAAVFIGLPVLAIVINVLSQLLLPKDPTLPPVVFHVIPWFG

SAAAYGMDPYKFLFDCREKYGDVFTFILLGRRMTVALGPKGNNLSLGGKITHVSAEEAYT

HLTTPVFGKGVVYDCPNDMLMQQKKFSRSRVCCFCRSASGSPMPRSTPARTFRRNPTNAQ

IKHGLTTEALSSYAELMRGETRQYFRDHVVTGKPFEVLEMMQQLIILTASRTLQGKEVRE

NLDIRFAKLLEDLDKGFTPVNFLFPNLPLPSYKRRDKAQKEMSDFYMSIIEKRRSGEHDH

ENDMIAALQGSVYKNGVPLSDRDISHIMIAILMAGSHTSSATSSWFLLHLAYDQELQQAL

YDEQVQLFGNADGSFREMTLEDTRELPLMTACIRETLRLHAPIHSIYRKVMLDIAVPPSL

AAPSKDGTYVIPKGHFIVAAPGVSQMDPRIWKDAKTWRPRRWLEEGGVANAANEEYTSGE

RVDYGFGAVSKGTESPYQPFGAGRHRCVGEQFAYLQLTMLVSEIIRTFKVEPGAPQFPET
NYQTMIVLPLHGMIKLEPRK

>CYP51F(345805) Naganishia vishniacii v1.0
MSSSRILSLLPLEQLSWASIAAIILVGLPALLVVGNIIRQKVLPKDPSLPPVVFHLIPWF
GSAATYGMDPYKFLFDCREQYGDLFTFVLLGRKMTVALGPKGNNLILGGRLNQVSAEEAY
THLTTPVFGEGVVYDCSNDLLMQQKRMVKFGLSTENLRAYVGMITEETLGFLTNELKASP
TAWQGFDALNAMSELTILTASRTLQGKEVRAGLDKTFAERYEHLDGGFTPINFMFPNLPL
PSYRRRDKAQKAMSEFYQGIIRKRREGTHDHEYDMISALQSSKYKDGTPLSDRDIAHMMI
ALLMAGQHTSSATSSWTLLHLAERTDIWEELYREQKNKFGNPDGTFRDLTYEELKELPVL
DHVIRETLRLHAPIHSIMRKVISDIPVPNTLSSPKQHENATYVIPKGHFVLASPGVAQMD
PLIWQNAEEWNPHRWDDESGVAAMAAEQYTEGDKVDYGYGSVSKGTESPYQPFGAGRHRC
IGESFAYVQLQTILATLCRRVVLKLEGPFPAPNYQTMIVLPLKGQTKIMYKLRA

>CYP51F(367271)Cryptococcus curvatus
MAMDPSAVLDWAHALPLPVKIVGAVIGVPLLVIVLNVLRQLIVPTDPTAPPVVFHYIPWF
GSAAYYGMDPYKFMFECRDKYGDVFTFILLGRRMTVALGPKGNNLSLGGKVSHVSAEDAY
THLTTPVFGKGVVYDCPNDMLMQQKKFIKHGLTTEALQSYASLMPAECHGYFNNELKITK
ANPGPKTVDVLHVMAELIVLTASRTLQGKEVRENMSIRFAKLLEDLDKGFTPLNFMFPNL
PLPSYRRRDLAQKEMSDFYMSIMAKRRTGEHDHEPDMIQALQGSVYRNGTPLSDRDIAHM
MIALTMAGQHTSSATSSWFLLHLAHDQDIQKRLYEEQVEYFGNPDGTFRPMTYEDTKKLP
LMDSCIRETLRLHAPIHSIYRKVLQPIVVPQSLAAPSEDKAYVIPKGNFIVAAPGVSQMD
PKIWDDAPRWNPLRWLVDGGIAKTANEQYQAGEKVDYGFGAVSKGTESPYQPFGAGRHRC
IGEQFAYLQLTLVVGEVVRNYKLTAAQREFPKTNYQTMIVLPLDPKITFEARV

>CYP51F1(38441)Tremella mesenterica Fries v1.0
MSLSIASVPWAPQWLLSLPSPLLVLLGIIGFPTLIIFLNVFRQLVLPRDKNLPPVVFHYI
PWFGSAAYYGEDPYKFYFECREKYGDVFTFVLLGRNITVALGPKGNNLSLGGKTTQVSAE
EAYKHVTTPVFGKGVVYDCPNEMLMQQKKFIKNGLSTEALASYPPLINAECEHYFEKELG
FSPTSPGPKTIEVFKIMSQLIILSASRTLQGKEVRESLDSKFAEYFHDLDGGFTPLNFMF
PNLPLPSYRKRDKAQKSMSDFYLNIMQKRREGATDVNGSFDMLAALQGCVYRNGVPLTDR
DIAHMMIAILMAGQHTSSATSSWALLHLAHRPDVADALYEEQKTLFGNPDGSFRPVEYGD
EKRMTLMNSIIRETLRIHAPIHSIYRKVISPMPVPASLSAPSESATYIIPKNYYIVAAPG
VSAMDPRIWKDANVWEPFRWSDEKGTAAMALNEYTTGGEKMDYGFGQVSKGTESPYQPFG
AGRHRCVGEAFAYVQLSVIIAYIIRNYSLRLETPDGLFPKTNYRTMIVLPLNGLMSLTKR
KQEV

>CYP51F1(523016) Naematella encephela UCDFST 68-887.2 v1.0

MTVALGPKGNNLSLGGKVSQVSAEDAYTHLTTPVFGKGVVYDCPNDLLMQQKKFIKHGLT
TEALSSYAPLMYKEATDFFEHELGLTPATPGPKKLEALKIMSELIILTAGRSLQGKEVRA
SLNARFASLYEDLDGGFTPLNFMFPNLPLPSYRRRDRAQKEMSDFYLDIIRKRREGETEP
EHDMISVLSGCVYRDGTPLTDRDVSHMMIAILMAGQHTSSATSSWILLHLAHRPDVVEAL
YEEQKKLFGNQDGTFREVTYESTKQMTLMEAIIRETLRLHAPIHSIYRKVLSDIPVPASL
SSPSESSAYVIPEGHFIVAAPGVSQMDPLIWPDAKTWNPYRWLDEKGVAASAKEQYSGAT
SEQVDYGFGQVSKGTESPYQPFGAGRHR

>CYP51F1(627561)Kockovaella imperatae NRRL Y-17943 v1.0
MDVFNNPFVANMMMNTGRGFNRQPTEAQREFARNFREGKIQPIDPTIPLYILSSIAILLF
TVVGSNLMQQFLAPRDPSHPPVVLHYFPWFGSAYSYGTDPYKFLFKCRAKYGDVFTFIMF
GRKMTVALGPKGNNLSLGGKVSHVSAEDAYTHLTTPVFGKGVVYDCPNEMLMQQKKFIKH
GLTPQAFQTYAPLIWQETHQFFEQEKGFNVYPGPKSYEAIKFFSELIILTASRTLQGKEV
RAGLTSSAAKHFEHLDKGFTPINFLFPNLPLPSYRKRDKAQKAMSDFYLEIMRKRREGES
DVDEHDMIAALTGNEYKDGTPLTDRDVAHMMIAILMAGQHTSSATSSWTLLHLAQKPLIF
KALFEEHRDLFQNEDGTWEDVTYESTKEMPLMAAVIRETLRMHAPIHSIYRKVLQDLEVP
PALAAGGNGSRPYVVPKGHFVVAAPGVSAMDPKVWPDADKWDPFRWLEDKGMAQEALDSY
SGATSEQIDYGFGQVSKGTESPYMPFGAGRHRCVGEQFAYLQLSVIMSYIVRNYDIQLLG
AFPQTNYNTMIVLPLNGYVTFKKRGQSKWILSTK

>CYP51F(629036)Cryptococcus werringae
MRLSTCLAVATAIVAPAKAIIQHPVSVYLLPSPDASPSTVKAPTLDGDEAFDVFANHFGV
GSQSTFEKYQKKSWAHLVDPSHSSELTGEEQAKVIIIQGDLSPSDILPPTYQDSLADFEL
PSFPSYETMNFLQPFMIRGQKVVGHVLGENQDGLEEGFDRVWSNVKGSQVGKKMASAFDL
NTSLHAQALHAELQALSALAMNLPVDARLERNRNVWDSVVISSLGTNDSTGVDAEVREMG
LNVIRASLQALAANPKQPRVVLVVVPTTSGAAFAPYVAAVKKFFTRLTTSSDQSILLGFL
PTLFSSFGTIGSVLLLLLSLPVLLVAGNVFRQKVLPRDPTLPPLVFHYIPWFGSAATYGM
DPYKFLFDCREQYGDLFTFILLGRKMTVALGPKGNNLILGGKLSQVSAEDAYTHLTTPVF
GKGVVYDCPNEMLMQQKRMVKFGLSTENFRVYVPLIKQETLDFLGKDLKPSNEWKGFNAL
HAMAELTILTASATLQGKEVRGKLDKTFAKRYEALDGGFTPINFMFPNLPLPSYKRRDKA
QAEMSEFYQSIIRGRREGASEHDHDMIAALMASTYRDGTPLSDSDIAHMMIALLMAGQHT
SSATSSWTLLHLADRTDVYAELYEEQKEKFGNPDGTFRDMTYEDLKDLPKLDSVIRETLR
MHAPIHSIMRKVMTDMPVPNALAAPTANEKSSYIVPKGHFVLASPGVAQMDPLIWSDASI
WNPHRWTDEKGMASQALEEYTSGDKVDYGYGSVSKGTESPYQPFGAGRHRCIGETFAYLQ
LQTILATFIRNVEMKLDGPFKTNYQTMVVLPLPGTKILYRNRK

>CYP51F1(809380)(Cryptococcus terricola)

MDPYKFMFGCRAKYGDLFTFILMGREMTVALGPKGNNLILGGKLSQVSAEEAYTHLTTPV

FGKGVVYDCPNDMLMQQKRMVKFGLSTENLKSYVPKITRETLDFLSHGLHTSATKWQSFN

ALDKLAELITLTASDCLQGREVRAGLDKTFAKRIEALDKGFTPMNFVFPNLPLPAYWNRD

KAQKEMSEFYQEIIRKRREGTHDHEHDMLEALMGSSYKNGTPLTDSDIAHMMIALLMAGQ

HTSSATSSWTLLHLAQRHDIYEALYQEQKEKFGNADGTFRDLTYEDLKDLPLLDSCIRET

LRLHAPLHSIMRKVISDIPVPNSLSSPSQTNDASYVIPKGHFLMACPGVSQMDPLIWQDS

TTWNPYRWSDERGVAAQAVEEYQGEKGEKVDYGFGSVSKGTESPYQPFGAGRHRCIGESF

AYVQLQTIIATVVRNLTLKFEGDFPSPNYQTMVVLPKGGEILFKSRQ

>CYP51F(KIR77383.1/E6QZS1)Cryptococcus gattii EJB2

MSAIIPQVQQLLGQLAQYIPPWFTALPTSLKIVIAVIGIPAFIIGLNVFQQLCLPRTKDL

PPVVFHYIPWFGSAAYYGEDPYKFLFECRDKYGDLFTFILMGRRITVALGPKGNNLSLGG

KISQVSAEEAYTHLTTPVFGKGVVYDCPNEMLMQQKKFIKSGLTTESLQSYPPMITSECE

DFFTKEVGISSQKPSATLDLLKSMSELIILTASRTLQGKEVRESLNGQFAKYYEDLDGGF

TPLNFMFPNLPLPSYRRRDEAQKAMSDFYLKIMENRRKGESDHEHDMIENLQGCKYRNGV

PLSDRDVAHIMIALLMAGQHTSSATSSWTLLHLADRPDIVEALYQEQKEKLGNPDGTFRD

YKYEDLKELPIMDSIIRETLRMHAPIHSIYRKVLSDIPVPPSLAAPSENGQYIIPKGHYI

MAAPGVSQMDPRIWQDAKVWNPARWHDEKGFAAAAMAQYTKAEQVDYGFGSVSKGTESPY

QPFGAGRHRCVGEQFAYTQLSTIFTYVVRNFTLKLAVPKFPETNYRTMIVQPTNPLVTFT

LRNAEVKQEV

>EAL23379

QVQQLLGQVAQFFPPWFAALPTSLKVAIAVVGIPALIIGLNVFQQLCLPRRKDLPPVVFHYIPWFGSAAYYGEDPYKFLF

ECRDKYGDLFTFILMGRRITVALGPKGNNLSLGGKISQVSAEEAYTHLTTPVFGKGVVYDCPNEMLMQQKKFIKSGLTTE

SLQSYPPMITSECEDFFTKEVGISPQKPSATLDLLKAMSELIILTASRTLQGKEVRESLNGQFAKYYEDLDGGFTPLNFM

FPNLPLPSYKRRDEAQKAMSDFYLKIMENRRKGESDHEHDMIENLQSCKYRNGVPLSDRDIAHIMIALLMAGQHTSSATS

SWTLLHLADRPDVVEALYQEQKQKLGNPDGTFRDYRYEDLKELPIMDSIIRETLRMHAPIHSIYRKVLSDIPVPPSLSAP

SENGQYIIPKGHYIMAAPGVSQMDPRIWQDAKVWNPARWHDEKGFAAAMVQYTKAEQVDYGFGSVSKGTESPYQPFGAG

RHRCVGEQFAYTQLSTIFTYVVRNFTLKLAVPKFPETNYRTMIVQPNNPLVTFTLRNAEVKQEV

CYP51-ligand groups

- **Models with clashes**

There is a small but noticeable number of clashing conformations observed for many CYP51F-ligand conformations, most likely artifacts of the modelling process. Its fraction ranges from nearly 0% (1 model in 120 000) for clotrimazole to 0.15% for itraconazole. Interestingly,

eburicol, lanosterol and obtusifoliol metabolites recorded a significantly higher fraction of incorrect conformations, above the average. These models could, in general, be easily excluded on an early stage of analysis, either by a filtering protocol in Rosetta itself, or by a BioShell script. Here we report them only to draw the complete analysis.

- **Correct conformations**

In this semi-global docking scenario as we applied in our study, ligands explored nearly the whole surface of a protein. The chance of finding a correct solution is therefore relatively low. Here we observe from 14 correct solutions for ketoconazole to 212 correct solutions for lanosterol - from 0.012% to 0.18% of all models generated for a given ligand. The dependence of these statistics on a protein is considerably weaker than on a ligand. The number of correct solutions ranges from 51 for KIR77383 to 143 cases for 523016 ortholog.

- **Nearly-correct conformations**

The 1Å range of d_{NFe} distance we assumed to define correct solutions is pretty tight. For more generous 2Å criterion a large fraction of ligand molecules are located correctly in the active site even though their interaction with an iron atom is not that close. Given such an extended criterion of success, for all 108 combinations "nearly-correct" solutions were found.

- **In the pocket conformations**

The fourth group consists of models where a small molecule agent still resides in the active pocket, but is quite far from the heme cofactor. Orientation of a ligand is very diverse in these models and often different from what might be expected from experimental data. Total energy for models in this group is comparable to the values observed for "correct", and "nearly-correct'' models. The main reason for this is because Rosetta is unaware of the specificity of the system we study. None of the two energy functions that was used by Rosetta modelling software: "soft" for docking and "hard-rep" for final scoring, contain a term that would explicitly assess interactions between a ligand and a heme cofactor, besides standard Van der Waals and Coulomb interactions. Most notably, there is no potential to mimic a semi covalent bond that is formed between a heme iron atom and an azole nitrogen. This interaction has significant contribution to the total energy of a complex. Including this effect in the modelling protocol would certainly favor the correct geometry. In the majority of models therefore an inhibitor is further away from heme that 8 Å and doesn't count to any of the four considered categories.

Table S1. Summary statistics of CYP51s from *Tremellomycetes* alignments with CYP51 from *Saccharomyces cerevisiae* (PDB code: 4LXJ)[1].

CYP51 name	Protein ID	Number of Amino acids in CYP51	Number of amino acids aligned with 4LXJ	E-value	Identities	Similarity
CYP51F CngH99	00040	549	523	7.4E-67	43.0%	78.6%
CYP51F1 TaaCBS2479	1742	562	507	3.5E-63	44.0%	76%
CYP51F1 Nvis	345805	536	514	4.7E-67	44.0%	78.2%
CYP51F Ccur	367271	535	512	6.5E-65	44.0%	78.5%
CYP51F1 Tmfri	38441	540	514	1.9E-65	41.0%	74.3%
CYP51F1 Nen UCDFST68-887	523016	390	364	3.8E-50	50.0%	89.3%
CYP51F1 Kim NRRL Y-17943	627561	548	525	7.5E-63	41.0%	75.7%
CYP51F Cwer	629036	533	516	1.6E-55	45.0%	80.3%
CYP51F Cter	809380	468	445	7.7E-62	46.0%	84.3%
CYP51F1 Cneo B-3501A	EAL23379	546	524	2.00E-66	41.0%	76.8%
CYP51F Cgat EJB2	KIR77383	549	523	1.5E-66	42.0%	78.3%

Table S2. Analysis of active site cavity amino acids in *Tremellomycetes* CYP51s. CYP51s represented with their protein IDs. The nature of active site cavity amino acid composition was calculated at the webserver https://www.peptide2.com/ using the Peptide Hydrophobicity/Hydrophilicity Analysis tool. Hydrogen bonds, stacking interactions and Van der Waals interactions are marked in blue, green and black font, respectively.

CYP51F	Active site cavity amino acids	No. of Amino acids in cavity	Cavity volume [Å3]	Nature of active site	Amino acids interacting with ligands								
					Clotrimazole	Eburicol	Fluconazole	Itraconazole	Ketoconazole	Lanosterol	Obtusifoliol	VT-1129	Voriconazole
00040	F71 S73 A74 A75 Y76 Y77 G78 E79 D80 P81 L100 M101 R103 L118 A127 A130 Y131 L134 T135 F139 V143 V144 Y145 M151 L152 M153 Q155 K156 K157 I159 K160 L163 A212 L216 F240 T241	99	1518	Hydrophobic: 54.55% Acidic: 3.03% Basic: 12.12% Neutral: 30.3%	Y131 HEM1 F139 F240 Y145 T135 T321 M316 M528 A313 I386 A317 V144	Y131 Y145 I389 A317 F240 V144 I386 A313 HEM F139 T321	W445 HEM Y480 F483 Q481 R377 A384 F245 Y145 A457 L380 R381 M528 A317 Y131 Q428 T321 W445 P482 E448 I386 P385	Y390 F240 F245 H387 Y131 P242 L100 Y390 HEM Y77 V144 I386 T321 F139 M528 G78 A317 T527	Y390 F240 F245 H387 Y131 P242 L100 Y390 HEM Y77 V144 I386 T321 F139 M528 G78 A317 T527	M528 A324 S322 F240 A317 L380 P385 V144 T325 I386 Y145 Y131 HEM H320 T321 T135	L134 A130 H133 F240 A317 F245 T321 M528 Y131 L100 Y390 HEM I386 Y145	Y131 F139 H320 F240 Y145 Q319 Y131 M316 T321 HEM I386 D237 A317 Y233 M528	I386 Y131 Y145 HEM F240 P385 T135 A317 G318 T321 I386 M528

	P242<br>L243<br>N244<br>F245<br>M246<br>M287<br>H309<br>I310<br>M311<br>I312<br>A313<br>L314<br>L315<br>M316<br>A317<br>G318<br>Q319<br>H320<br>T321<br>S322<br>S323<br>T325<br>L380<br>A384<br>P385<br>I386<br>H387<br>S388<br>I389<br>Y390<br>R391<br>A422<br>P424<br>G425<br>F451<br>A452<br>A453<br>A454<br>Q481<br>P482<br>F483<br>G484<br>A485<br>G486<br>R487<br>H488<br>R489<br>C490												

	V491<br>G492<br>E493<br>Q494<br>F495<br>A496<br>Y497<br>Q499<br>L500<br>N524<br>Y525<br>R526<br>T527<br>M528<br>I529												
1742	W58<br>F59 S61<br>A62<br>A63<br>A64<br>Y65<br>G66<br>M67<br>D68 P69<br>F86 L88<br>L89<br>M93<br>C155<br>R156<br>T258<br>P259<br>V260<br>N261<br>F262<br>L263<br>F264<br>Y272<br>H404<br>S405<br>Y407<br>N541<br>Y542<br>Q543<br>T544	32	685	Hydrophobic: 50%<br>Acidic: 3.13%<br>Basic: 6.25%<br>Neutral: 40.63%	HEM1<br>S159 F257<br>N137 Y133<br>I403 P402<br>T544 HEM1<br>A334 T338<br>M333 H337<br>I406 A158	F257<br>Y119<br>F127<br>I403<br>F257<br>T258<br>A158<br>S159<br>M545<br>T544<br>S157<br>F262<br>A334<br>T338<br>G160<br>L122<br>HEM1<br>R156<br>P259<br>V126<br>T123	HEM1 S159<br>F127 F257<br>T544 G160<br>M545 I403<br>T338 P402<br>T123 A334<br>S157 A158	I110 S157<br>S159<br>Y484 H505<br>Y119<br>A158 T338<br>I406 R408<br>P136 G108<br>Y407 I403<br>N137 R504<br>S405 L106<br>I110 S105<br>F257 M545<br>K109 HEM1<br>G107	F257 H337<br>F127<br>T123 F262<br>I226 A334<br>M333 T338<br>D254 M545<br>L122 T544<br>S157 F257<br>S336 HEM1<br>A158 R156<br>S159 G160<br>Y119 L250	V126<br>G335 S339<br>A158<br>HEM1<br>H337 F127<br>T338 M545<br>A334 A513<br>F257 T342<br>A330 Y133<br>I403 S157<br>P402 M333<br>S159	G160 T123<br>I403 T338<br>L122 P402<br>Y119 S159<br>M333 G335<br>F257 S157<br>HEM1 H121<br>V126 A334<br>P125 T124<br>A158 P162<br>T120 F127<br>M545	A158<br>H337<br>F257<br>Y407<br>F262<br>F127<br>R156<br>M333<br>A334<br>M545<br>HEM1<br>S157<br>L122<br>T123<br>A158<br>V126<br>T338<br>T544	H337 I546<br>T338<br>HEM1 F257<br>F127 Y133<br>P402 T544<br>A330 A334<br>HEM1 S157<br>R156 H337<br>M545 I403<br>M333 A158
345805	S62 A63<br>A64<br>T65	98	1696	Hydrophobic: 56.12%<br>Acidic: 2.04%		T309<br>H308							F485 A486<br>E483

	Y66<br>G67<br>M68<br>D69 P70<br>F87 L89<br>L90<br>M94<br>L107<br>A116<br>A119<br>Y120<br>L123<br>T124<br>F128<br>V133<br>Y134<br>N138<br>L141<br>M142<br>Q144<br>K145<br>V148<br>L152<br>T196<br>A200<br>L204<br>F228<br>T229<br>P230<br>I231<br>N232<br>F233<br>M234<br>F235<br>M275<br>M298<br>I300<br>A301<br>L302<br>L303<br>M304<br>A305<br>G306<br>Q307<br>H308<br>T309<br>S310<br>S311			Basic: 8.16%<br>Neutral: 33.67%	HEM1 Y120 F228 F128 H308<br><br>V133 M304 T309 HEM1 I374 V519 A301 Y134 A305 I518	F228<br>Y120<br><br>Y120<br>T124<br>P373<br>S310<br>L123<br>HEM1<br>Y134<br>F228<br>A305<br>I374<br>F128<br>M304<br>T309<br>I377	A301 F128 T309 F228 V133 I518 Y120 Y134 A305 HEM1	Y134 F128 HEM1<br><br>L123 L140 F228 V133 Q143 P289 V132 C136 L141 Q144 T288 M147 I518 K285 T309 L290 M298 Y284 K145 A305 K283 Y134 I374	F128 H308 F228<br><br>S193 Q307 D225 L123 A305 HEM1 Y221 T309 Y120 M304 T124 I197	H308<br>S310 T309 F228 Y120 A305 F485 V519 T196 Q489 Y134 Q307 L303 I374 G306 HEM1	Q307 Y134 A305 G306 L302 Y120 L123 I374 HEM1 F128 F228 T124 M304 L303 L204 T309	Y120<br>F233<br>Y134<br>F128<br>F228<br><br>I518<br>M517<br>HEM1<br>I374<br>T309<br>T124<br>A305<br>M378<br>P230	F473 Y487 Y470 HEM1<br><br>I481 C480 A442 V441 L368 P472 F485 F473 S484 G482

	T313<br>I364<br>L368<br>H371<br>A372<br>P373<br>I374<br>H375<br>S376<br>I377<br>M378<br>R379<br>L411<br>P414<br>G415<br>Q418<br>V441<br>Y470<br>Q471<br>P472<br>F473<br>G474<br>A475<br>G476<br>R477<br>H478<br>R479<br>C480<br>I481<br>G482<br>E483<br>S484<br>F485<br>A486<br>Y487<br>Q489<br>L490<br>Y514<br>Q515<br>T516<br>M517<br>I518<br>V519<br>L520												
367271	F60 S62<br>A63<br>A64<br>Y65	115	1873	Hydrophobic: 52.17%<br>Acidic: 1.74%<br>Basic: 12.17%					HEM1 H310		T311	H377<br>Y380	

	Y66 G67 M68 P70 F87 L89 L90 R92 M94 L107 A116 A119 Y120 L123 T124 F128 V132 V133 Y134 M140 L141 M142 Q143 Q144 K145 K146 F147 I148 K149 H150 G151 L152 I198 A202 L206 F230 T231 P232 L233 N234 F235 M236 F237 M277 H299 M300 M301 I302 A303 L304			Neutral: 33.91%	Y134 F128 HEM1 F230 Y120<br><br>A303 A307 M518 I376 M306 H310 T311	Y134 Y120<br><br>Y120 A119 A116 D118 E117 T124 M518 I376 HEM1 Y134 T311 T121 A307 F230	L123 Y120 A307 HEM1 I376 M518 T311 F230 T517	Y66 Y380 F87 HEM1 H377<br><br>P414 M518 P375 S378 F230 M74 F85 T124 V96 I376 A412 F73 I379 Y66 C77 V411 A413 F128 L123 A307 T311 L98 V416	M306 G308 T517 S312 T201 T311 L206 F230 M518 Q309 I376 HEM1 A307 Q489 A202 I198 Y120	G308 F128 T311 A307 F230 Y134 V133 A303 H310 I376 M518 T124 HEM1 Y120 P375	P375 Y134 I376 A307 M518 F230 A119 F235 Y120 L123 H122 T311 R92 HEM1	F235 F128 H310 F230<br><br>A307 L123 M518 HEM1 P232 T124 I376 M306 T311 V127 T517	F230 HEM1 Y134 F128 Y120<br><br>T311 M518 F230 A307 A116 T124 I376 R381

	T305<br>M306<br>A307<br>G308<br>Q309<br>H310<br>T311<br>S312<br>S313<br>T315<br>S316<br>D363<br>I366<br>R367<br>L370<br>H373<br>A374<br>P375<br>I376<br>H377<br>S378<br>I379<br>Y380<br>R381<br>A412<br>P414<br>G415<br>Q418<br>I441<br>A442<br>G458<br>F459<br>G460<br>Y470<br>Q471<br>P472<br>F473<br>G474<br>A475<br>G476<br>R477<br>H478<br>R479<br>C480<br>I481<br>G482<br>E483<br>Q484												

	F485<br>A486<br>Y487<br>L488<br>Q489<br>L490<br>N514<br>Y515<br>Q516<br>T517<br>M518<br>I519												
38441	F58 S60<br>A61<br>A62<br>Y63<br>Y64<br>G65<br>E66<br>D67 P68<br>L87 L88<br>R90 I92<br>N101<br>L105<br>A114<br>A117<br>Y118<br>K119<br>V121<br>T122<br>F126<br>V130<br>V131<br>Y132<br>N136<br>L139<br>M140<br>Q142<br>K143<br>I146<br>K147<br>G149<br>L150<br>I196<br>S199<br>A200<br>S201<br>T203	107	1762	Hydrophobic:<br>50.47%<br>Acidic: 3.74%<br>Basic: 11.21%<br>Neutral:<br>34.58%	F126 Y118<br>H310 HEM1<br>F228<br><br>V131 A307<br>Y132 F126<br>T311 M306<br>I522 A303<br>I376	Y118<br>F228<br>F126<br><br>F233<br>T122<br>I522<br>V121<br>I376<br>Y132<br>H310<br>P230<br>HEM1<br>M306<br>T311<br>A307<br>V125<br>T229<br>M521<br>G227	HEM1<br>Y118 F126<br>T311 M521<br>A307 T122<br>F228 I376<br>Y132	H310 H222<br>F228<br><br>Y118 T520<br>A307 D225<br>M521 I522<br>V131 HEM1<br>L524 I376<br>Y132 T311	F228 H310<br><br>HEM1 I376<br>V131 T122<br>F126 M521<br>Y132 M306<br>D225 Q309<br>A307 L524	G308<br>F126 A307<br>S312 Y118<br>Q309 I376<br>I522 P375<br>V131 F228<br>T122 Y132<br>I196 T311<br>HEM1	F228 H377<br>G308 S378<br>Y380 F233<br>Y132 P230<br>A307 M306<br>T311 A303<br>F126 I376<br>Y118 HEM1<br>M521 I379	E115<br>A114<br>Y118<br>F126<br>Y132<br>H479<br><br>S113<br>Y118<br>A307<br>L139<br>HEM1<br>T109<br>R381<br>E115<br>N136<br>A303<br>V131<br>I376<br>R478<br>T311	A307<br>Y132 Y118<br>HEM1 F126<br><br>G308 I376<br>R381 A307<br>A303 F228<br>T311 Y132

	L204<br>D225<br>G226<br>F228<br>T229<br>P230<br>L231<br>N232<br>F233<br>M234<br>F235<br>M277<br>M300<br>I302<br>A303<br>I304<br>L305<br>M306<br>A307<br>G308<br>Q309<br>H310<br>T311<br>S312<br>S313<br>T315<br>L370<br>H373<br>A374<br>P375<br>I376<br>H377<br>S378<br>I379<br>Y380<br>R381<br>A412<br>P414<br>G415<br>F460<br>Q462<br>Q472<br>P473<br>F474<br>G475<br>A476<br>G477<br>R478												

	H479 R480 C481 V482 G483 E484 A485 F486 A487 Y488 Q490 L491 N517 Y518 R519 T520 M521 I522 V523												
523016	M1 L14 A23 D25 A26 Y27 T28 H29 L30 T31 V34 F35 V39 V40 Y41 L47 L48 M49 Q51 K52 K53 F54 I55 K56 H57 G58 L59 T60 T61 A63 L64 S65 Y67 A68 P69 L70 M71 Y72	157	4173	Hydrophobic: 49.68% Acidic: 5.73% Basic: 11.46% Neutral: 33.12%	HEM F35 Y41 Y27 F137 Y27 HEM1 I283 V40 H217 Y41 A214 T218 F35	H217 F137 F35 Y27 T218 V40 HEM1 A214 L48 S219 P282 I283 G215 Y41 T31	T31 Y27 I283 F35 A210 M213 A214 F137 Y41 HEM1 L30 T218	N45 Y27 H387 F35 Y41 HEM1 I283 Y27 N45 F137 V40 A214 L48 T218 V18 E24 A210 P44 C43 A23	E354 Q355 A352 R388 K353 K56 HEM1 S351 F382 L59	G36 V40 Y41 A214 H206 T32 M207 F35 T28 I283 T218 T31 V39 A210 Y27 F137 HEM1 H217	F35 HEM1 T218 T222 I283 V40 A214 L277 Y41 H217 A281 A210 S219 T31 P282	F137 Y41 Y27 H217 D134 T218 HEM1 F137 A214 F35 I283	H217 F137 HEM1 F35 Y27 Y41 A214 T31 T218 I283

	K73<br>E74<br>A75<br>L98<br>M101<br>S102<br>L104<br>I105<br>I106<br>L107<br>T108<br>A109<br>G110<br>S112<br>L113<br>Q114<br>Y130<br>E131<br>D132<br>L133<br>D134<br>G135<br>G136<br>F137<br>T138<br>P139<br>N141<br>F142<br>M143<br>F144<br>P145<br>D183<br>H206<br>M207<br>M208<br>I209<br>A210<br>I211<br>L212<br>M213<br>A214<br>G215<br>Q216<br>H217<br>T218<br>S219<br>S220<br>A221												

	T222<br>S223<br>S224<br>W225<br>I226<br>L227<br>L228<br>L230<br>L240<br>Q244<br>F248<br>T254<br>F255<br>V258<br>T259<br>Y260<br>E261<br>S262<br>T263<br>K264<br>Q265<br>M266<br>M269<br>E270<br>I272<br>I273<br>R274<br>L277<br>H280<br>A281<br>P282<br>I283<br>H284<br>S285<br>I286<br>Y287<br>R288<br>K289<br>F316<br>I317<br>V318<br>P321<br>W342<br>G347<br>V348<br>A349<br>A350<br>S351												

	A352<br>K353<br>E354<br>Q355<br>Y356<br>S357<br>G358<br>Y366<br>F368<br>G369<br>Q370<br>V371<br>S372<br>Y379<br>Q380<br>P381<br>F382<br>G383<br>A384<br>G385<br>R386<br>H387<br>R388												
627561	V59<br>W65<br>F66 S68<br>A69<br>Y70 S71<br>Y72<br>G73<br>T74<br>D75 P76<br>Y77<br>K78 F79<br>L80 F81<br>C83 F91<br>F93 I94<br>M95<br>F96 R98<br>K99<br>M100<br>T101<br>V102<br>L104<br>A125<br>Y126<br>L129<br>T130	61	1839	Hydrophobic: 55.74%<br>Acidic: 1.64%<br>Basic: 9.84%<br>Neutral: 32.79%	Y126 F134 HEM1 F235<br><br>M526 A309 T317 I382 T525 A313 M312	F235 Y126<br><br>P237<br>Y140<br>M526<br>F235<br>G314<br>T317<br>L129<br>M312<br>A313<br>I382<br>HEM1<br>H316<br>P381<br>T130<br>F240<br>Y386	T317 Y126 H316 T130 F235 I382 L129 A313 HEM1 T525	F235 H316<br><br>H316 T525 T317 Y386 S200 L129 E201 Q315 F240 M312 F235 Y126 D232 A313 E229 HEM1 I382 M526 I204	H379<br><br>N522 T521 M378 P381 I527 L129 H379 HEM1 T317 A320 T525 L529 V528 W324 I382 F235 H316 M526 A313	Q315 HEM1 I382 T130 Y140 Y126 I310 T317 P381 F235 A313 V139 G314 F134 S318 M526 L311	F494 A313 G314 Y140 I382 F235 S318 T317 Y126 H316 Q498 HEM1 A495 M526 I203	Y386 F240 F235 Y140 F134 Y126<br><br>V139 A309 HEM1 L129 I382 N524 H383 T525 P237 A313 T130	Y140 F235 HEM1 Y126<br><br>M526 T130 Y140 L129 I385 I382 T317 A313

	Y140<br>F235<br>T236<br>P237<br>I238<br>N239<br>F240<br>L241<br>F242<br>H316<br>P381<br>I382<br>H383<br>S384<br>I385<br>Y386<br>R387<br>V418<br>A419<br>A420<br>P421<br>V423<br>N522<br>Y523<br>N524<br>T525<br>M526<br>I527												
629036	A63<br>Y66<br>G67<br>M68<br>D69 P70<br>F87 L89<br>L90 R92<br>M94<br>L107<br>A116<br>A119<br>Y120<br>H122<br>L123<br>T124<br>F128<br>V132<br>V133<br>Y134	110	2123	Hydrophobic: 60%<br>Acidic: 4.55%<br>Basic: 9.09%<br>Neutral: 26.36%	HEM1 H477 Y458<br>A474 V461 F472 I480 C479 G481 R478 G473 HEM1 K145	A304<br>Y120<br>T124<br>S375<br>L141<br>G305<br>I373<br>A300<br>I376<br>HEM1<br>Y134<br>F128<br>V133	F227 HEM1 T124 F128 A304 L123 I373	F472 HEM1 Y469 W434<br>Y448 E437 F472 L367 Y120 R368 R364 V516 I373 P372 Q417 A371 A304 T308 P471 F227 Q470	F227 H307 F128<br>L123 M303 D224 A304 Y134 L302 I373 V127 Y120 F232 Q306 F227 I299 T124 M377 Y220 L223	Y120 L203 F227 S375 I376 Y134 F128 A300 Q306 A304 L301 G305 T308 I373 HEM1 L302 M303	H122 F232 S375 M377 I373 Y120 A304 HEM1 I376 A119 T308	Y120<br>F227<br>Y134<br>F128<br>F232<br>HEM1<br>A304<br>S375<br>A300<br>V133<br>Y66<br>T124<br>T308<br>I373	HEM1 Y134 Y120 F227<br>S375 HEM1 A304 T308 T124 I373 I376

	L141<br>Q144<br>K145<br>V148<br>L152<br>F157<br>L188<br>A192<br>T195<br>A199<br>L203<br>E221<br>A222<br>L223<br>D224<br>G225<br>G226<br>F227<br>T228<br>P229<br>I230<br>N231<br>F232<br>M233<br>F234<br>M274<br>M297<br>M298<br>I299<br>A300<br>L301<br>L302<br>M303<br>A304<br>G305<br>Q306<br>H307<br>T308<br>S309<br>S310<br>A311<br>T312<br>I363<br>L367<br>H370<br>A371<br>P372<br>I373					L301<br>T308<br>H374							

	H374<br>S375<br>I376<br>M377<br>R378<br>F408<br>V409<br>L410<br>A411<br>S412<br>P413<br>G414<br>Q417<br>E446<br>Q470<br>P471<br>F472<br>G473<br>A474<br>R476<br>H477<br>R478<br>C479<br>I480<br>G481<br>E482<br>T483<br>F484<br>A485<br>Y486<br>Q488<br>L489<br>T514<br>M515<br>V516<br>V517<br>L518<br>P519<br>L520<br>P521												
809380	D1 P2<br>Y3 F5<br>M6 F19<br>L21<br>M22<br>M26<br>V28	110	1769	Hydrophobic: 57.27%<br>Acidic: 2.73%<br>Basic: 7.27%<br>Neutral: 32.73%	F60 Y52 Y66 F160 HEM1<br>M236 A233 I309 T450	Y52 F60<br>T241 E49 V65 T56 Y66	M451 F60 A237 F160 L306 L55	F165 F19 H240 Y52 H307 F160	F160 F60 H240<br>A125 Q239 M310 D157	S242 T56 H240 HEM1	A237 L234 M451 HEM1 M236 H307	H240 F160 Y52	F165 HEM1 H240 Y52 Y66 F160

	L39<br>A48<br>A51<br>Y52<br>L55 T56<br>F60 V64<br>V65<br>Y66<br>N70<br>L73<br>M74<br>Q76<br>K77<br>V80<br>L84<br>N88<br>L89<br>Y92<br>V93 I96<br>L127<br>I128<br>T129<br>L130<br>T131<br>A132<br>S133<br>D134<br>C135<br>L136<br>Q137<br>F160<br>T161<br>P162<br>M163<br>N164<br>F165<br>V166<br>M207<br>L208<br>M230<br>M231<br>I232<br>A233<br>L234<br>L235<br>M236<br>A237<br>G238				M451 A237<br>T241 H240<br>L306	L306<br>F160<br>Y52 C68<br>HEM1<br>A237<br>T53 A48<br>M451	T241 Y66<br>V65 T56<br>HEM1 A233	HEM1 P2<br>L55 L21<br>A237 P162<br>F5 Y66 T161<br>F60 T450<br>M451 L306<br>Q449	Y66 HEM1<br>A237 T241<br>L306 Y52<br>F160 M236<br>M451 I153	A237 Y66<br>Q239 Q423<br>G238 F160<br>I128 T241<br>Y52 M451<br>F419	F165 M310<br>S308 F160<br>Y52 T56<br>L306 L55<br>A233 G238<br>Y66 F60	T450<br>HEM1<br>D157<br>A237<br>L306<br>T56<br>M236<br>H240<br>M451<br>L55<br>F60<br>T241<br>T161<br>Q449	L55 A237<br>T56 L306<br>M236 F60<br>M451 T241

	Q239<br>H240<br>T241<br>S242<br>S243<br>T245<br>I296<br>L300<br>H303<br>A304<br>P305<br>L306<br>H307<br>S308<br>I309<br>M310<br>R311<br>M343<br>C345<br>P346<br>G347<br>V373<br>Q405<br>P406<br>F407<br>G408<br>A409<br>G410<br>R411<br>H412<br>R413<br>C414<br>I415<br>G416<br>E417<br>S418<br>F419<br>A420<br>Y421<br>V422<br>Q423<br>L424<br>N447<br>Y448<br>Q449<br>T450<br>M451<br>V452												

	V453												
EAL23379	S67 A68 A69 Y70 Y71 G72 E73 P75 F92 L94 M95 I99 L112 A121 A124 Y125 L128 T129 V132 F133 V137 V138 Y139 N143 M145 L146 M147 Q148 Q149 K150 K151 I153 G156 L157 I202 A206 S207 L210 F234 T235 P236 L237 N238 F239 M240 M281 K289 H303 I304 M305	104	1566	Hydrophobic: 53.85% Acidic: 1.92% Basic: 10.58% Neutral: 33.65%	F133 F234 HEM1 Y125 F234 A307 H314 I380 I523 M310 A311 T315 F133 T129 I383	A378 H377 A311 I380 Y139 T129 T315 V138 A318 T319 P379 HEM1 F133 Y125 F234 H314 V524 L374	T315 T129 HEM1 I380 Y139 M310 F133 I523 A311 F234 H314	F234 H314 I523 A311 V138 V524 Y139 T235 HEM1 T315 M522 D231 Y125	HEM1 Y139 F133 I380 A311 Y384 L308 L146 I153 T129 F239 A307 Y125 F234 T315 Q149 HEM1 I304 V138	H314 S316 T315 L128 A490 V524 I380 G312 M310 Q313 HEM1 Q493 Y139 I523 T129 Y125 A311 P379 F234	T315 M310 A124 G312 F234 A311 Y125 H314 Y384 V524 HEM1 I380 T315 F239 I523 S382	Y125 Y139 Y384 F239 F234 T129 I523 Y125 L128 T315 HEM1 R520 A311 Y71 I380 G72	Y125 H314 HEM1 F234 L128 F133 A311 T315 Y125 V524 M310 I380 I523

	I306<br>A307<br>L308<br>L309<br>M310<br>A311<br>G312<br>Q313<br>H314<br>T315<br>S316<br>S317<br>T319<br>S320<br>I370<br>L374<br>A378<br>P379<br>I380<br>H381<br>S382<br>I383<br>Y384<br>R385<br>P418<br>A446<br>V451<br>Y474<br>Q475<br>P476<br>F477<br>G478<br>A479<br>G480<br>R481<br>H482<br>R483<br>C484<br>V485<br>G486<br>E487<br>Q488<br>F489<br>A490<br>Y491<br>Q493<br>L494<br>Y519												

	R520<br>T521<br>M522<br>I523<br>V524<br>Q525												
KIR77383	S73 A74<br>A75<br>Y76<br>Y77<br>G78<br>E79<br>D80 P81<br>F98<br>L100<br>M101<br>I105<br>L118<br>A127<br>A130<br>Y131<br>H133<br>L134<br>T135<br>F139<br>V143<br>V144<br>Y145<br>N149<br>M151<br>L152<br>M153<br>Q154<br>Q155<br>K156<br>K157<br>I159<br>K160<br>S161<br>G162<br>L163<br>T164<br>S167<br>L168<br>Y171<br>I208<br>I209	111	1662	Hydrophobic: 50.45%<br>Acidic: 2.7%<br>Basic: 11.71%<br>Neutral: 35.14%	Y145 HEM1 Y131 F240 F139<br><br>V144 T135 Y145 T321 H320 A317 I386 M316	Y131 F240 Y145<br><br>T136 F139 L134 M316 Y131 G140 T321 M528 A317 V143 I386 HEM1 D146 T135 A313 T132 V144	HEM1 F139 Y145 F240 A317 L134 I386 M528 M316 T321 Y131	HEM1 Y145 F139 F158<br><br>I310 A313 A317 L302 I386 L314 HEM1 I288 K295 M311 Y390 Q155 L152 M273 Y131 T321 I159 M528 F240 Q292 V307 V144 C294 L291 K156	H320 F240 F139<br><br>F240 M528 HEM1 M316 H320 I389 T321 Y233 I209 Q319 I386 E234 Y390 D237 Y131 A317	T135 S322 Y131 F495 A317 F240 HEM1 Q499 F139 I208 M528 L315 Q319 I386 T321 Y145 G318 H320	HEM1 I386 Y131 S322 S388 G318 Y390 Y145 M528 A317 L315 A313 L134 F240 F139 Q319 L314 T321 M316	Y145<br>Y131 F139 F245 Y390 F240 Y145<br><br>L134 Y131 HEM1 R103 I386 A317 T135 S388 T527 P242 M528	Y145 HEM1 F240 Y131<br><br>A317 T321 F139 Y145 T135 I386 T527 P385 M528

	T211<br>A212<br>S213<br>L216<br>F240<br>T241<br>P242<br>L243<br>N244<br>F245<br>M246<br>F247<br>M287<br>H309<br>I310<br>M311<br>I312<br>A313<br>L314<br>L315<br>M316<br>A317<br>G318<br>Q319<br>H320<br>T321<br>S322<br>S323<br>T325<br>I376<br>L380<br>H383<br>A384<br>P385<br>I386<br>H387<br>S388<br>I389<br>Y390<br>R391<br>P424<br>F451<br>Y480<br>Q481<br>P482<br>F483<br>G484<br>A485												

	G486<br>R487<br>H488<br>R489<br>C490<br>V491<br>G492<br>E493<br>Q494<br>F495<br>A496<br>Y497<br>Q499<br>L500<br>N524<br>Y525<br>R526<br>T527<br>M528<br>I529												

Table S3. List of amino acid residues with unfavorable conformations indicated by MolProbity [cite] in modelled CYP51 structures.

Protein ID	Residue No.
00040	25 A, 144 V, 193 K, 194 P, 351 L, 4 P, 450 G, 530 V
1742	19 K, 26 G, 138 D, 141 M, 153 C, 157 S, 158 A, 161 S, 176 P, 177 T, 213 K, 296 G, 297 E, 312 V, 374 S, 421 A, 422 A, 424 S, 474 E, 505 H, 506 R
345805	19 S, 133 V, 179 S, 291 S, 393 S, 445 A, 446 A, 452 G, 510 P, 517 M, 520 L
367271	14 A, 42 I, 44 P, 133 V, 285 V, 344 P, 437 V, 446 N, 449 Y, 510 F
38441	56 P, 131 V, 183 P, 267 G, 268 A, 272 N, 283 G, 285 V, 344 P, 345 D, 398 E, 445 A, 454 E, 460 F, 465 K, 509 P, 513 F, 523 V
523016	40 V, 84 G, 86 T, 88 A, 89 T, 151 S, 180 P, 254 T, 304 S, 351 S, 374 G
627561	14 G, 25 L, 49 A, 139 V, 429 K, 519 P, 524 N
629036	5 L, 6 G, 18 I, 41 K, 42 V, 133 V, 178 P, 266 G, 268 S, 341 P, 392 A, 506 G, 517 V
809380	65 V, 111 S, 325 S, 333 S, 444 P, 446 P, 448 Y, 449 Q
EAL23379	7 G, 24 L, 138 V, 348 P, 363 L, 399 A, 444 G, 525 Q, 530 L
KIR77383	26 L, 144 V, 186 E, 187 V, 188 G, 189 I, 354 P, 407 S, 432 R, 456 M, 530 V

Figure S1. Chemical structures of the ligands used in the study.

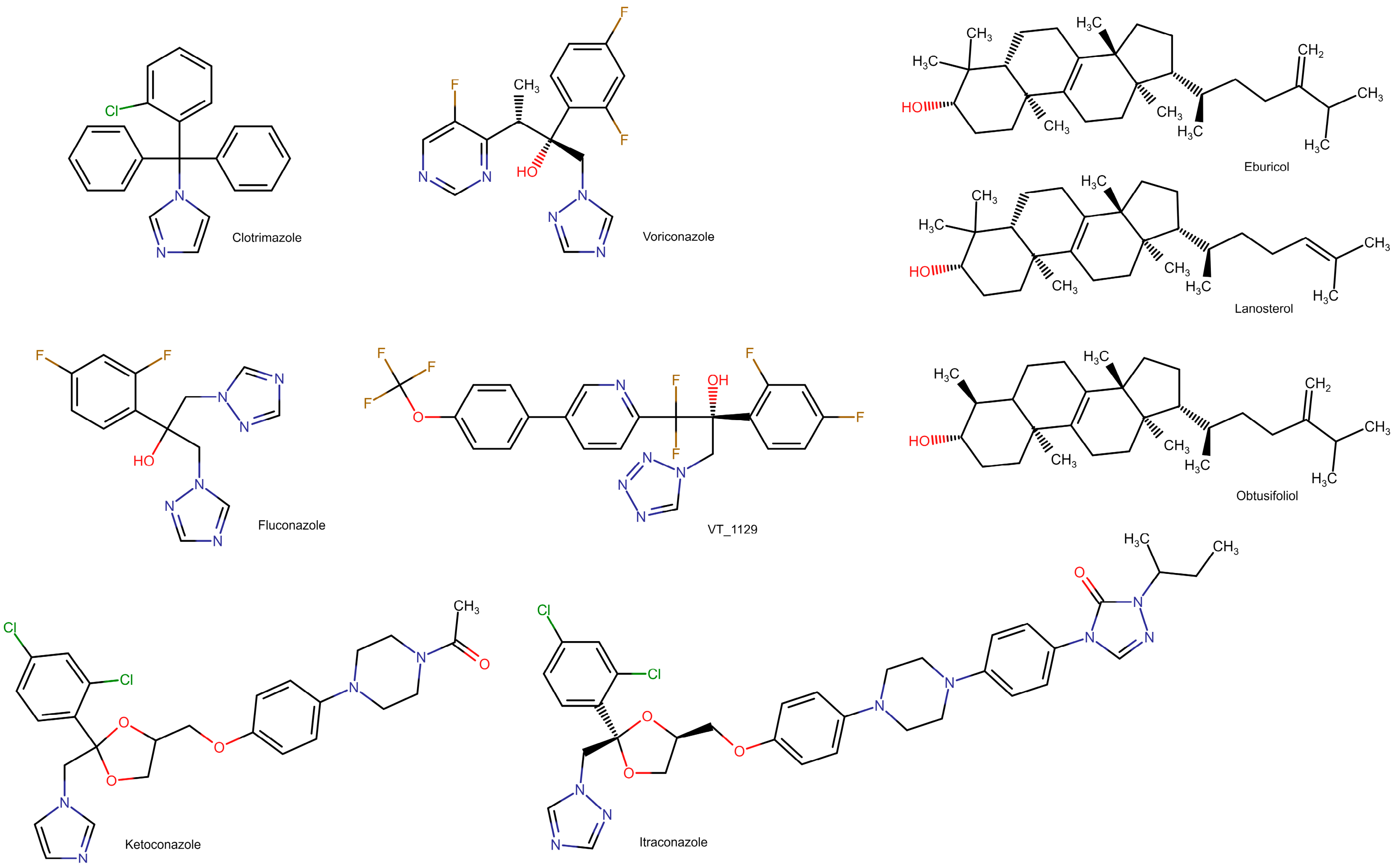

Reference

1 Monk, B. C. et al. Architecture of a single membrane spanning cytochrome P450 suggests constraints that orient the catalytic domain relative to a bilayer. Proceedings of the National Academy of Sciences 111, 3865-3870 (2014).
